# Whole-genome sequencing of 197 cases with Parkinson’s disease reveals novel pathogenic variants in the Indian population

**DOI:** 10.1038/s41531-026-01462-0

**Published:** 2026-07-06

**Authors:** Hiba Ali, Banashree Mondal, Andie Pinto, Camellia Sarkar, Bikram Kumar Panda, Khader Valli Rupanagudi, Vikram V. Holla, Tarunya Nagaraj, Shubha GS Bhat, Shweta Prasad, Mahima Bhardwaj, Pooja Mailankody, Rohan R. Mahale, Nitish Kamble, Ravi Yadav, Pramod Kumar Pal, Albert Stezin, Latha Diwakar, Shweta Ramdas

**Affiliations:** 1 Centre for Brain Research (CBR), Indian Institute of Science, Bengaluru, India; 2 Computational and Data Science Department (CDS), Indian Institute of Science, Bengaluru, India; 3 National Institute of Mental Health and Neurosciences (NIMHANS), Bengaluru, India

**Keywords:** Diseases, Genetics, Neurology, Neuroscience

## Abstract

Most insights into the genetics of Parkinson’s disease (PD) arise from European cohorts, leaving the genomic diversity of India underexplored. To address this gap, we performed whole-genome sequencing of 197 Indian PD cases (106 with early-onset PD (EOPD) and 91 with late-onset PD (LOPD)), capturing SNVs, indels, splice, and structural variants. We identified pathogenic variants in 31 cases across seven PD-associated genes (*GBA1*, *PRKN*, *PINK1*, *DJ1, SYNJ1, SNCA*, and *PLA2G6*), giving a diagnostic rate of 27.6% in EOPD and 2.2% in LOPD. Seven of these variants were novel to Indian populations. *GBA1* and *PRKN* were the most frequently mutated genes. Candidate genes with pathogenic variants were enriched in lysosomal pathways. Cases had significantly higher polygenic risk scores, supporting a polygenic contribution to PD risk. Our study establishes one of the first comprehensive sequencing-based resources for PD genetics in India, and underscores the need for large-scale genomic studies across diverse populations.

## Introduction

Parkinson’s disease (PD) is a complex neurodegenerative disorder clinically characterized by bradykinesia, rigidity, and resting tremor along with non-motor symptoms such as neuropsychiatric involvement, autonomic dysfunction, sleep disturbances, and olfactory loss^1^. The pathological hallmarks of PD include dopaminergic neuronal loss in the substantia nigra, and intracellular accumulation of α-synuclein aggregates known as Lewy bodies^2^. The global burden of PD has increased substantially in recent decades, with more than six million people affected in 2015 and projections suggesting over 12 million cases by 2040^3^. This steep rise, driven by aging populations and improved diagnostics, has led some to describe PD as a “pandemic”^4^. The mounting public health burden highlights an urgent need to understand its etiology across ancestrally and geographically diverse populations.

Genetic studies have greatly advanced our understanding of PD, with discoveries of both monogenic forms and polygenic contributions to disease risk^5^. 10–15% of PD cases have a family history, with 5–10% showing a clear Mendelian inheritance pattern; most cases (85–90%) are sporadic^6^. The most common monogenic PD-related genes are *SNCA, LRRK2, PRKN, PINK1, DJ1, GBA1*^7–9^. Variants in *GBA1* and *LRRK2* are among the most frequent risk variants worldwide (1–10% in different cohorts), with some ethnicity-specific hotspots^9–11^. Apart from Mendelian variants, many common variants of small effect contribute to PD, with individual alleles conferring either increased risk or protective effects, as revealed by genome-wide association studies (GWAS)^5,10^.

While rare variants in genes like *SNCA*, *LRRK2*, and *PRKN* cause familial PD^11^, recent meta-analyses have identified 90 risk loci explaining 20–30% of risk^10^. These findings have led to the development of polygenic risk scores (PRS), which aggregate the effects of both risk-increasing and risk-reducing alleles to stratify individuals according to their overall genetic risk. Apart from the genetic risk, environmental influences like pesticide exposure, metals, solvents, and even lifestyle factors affect PD risk^12–15^.

Until recently, most genetic studies have focused on individuals of European ancestry^16^. Over 80% of PD GWAS participants are of European descent^17^, and there are few large-scale studies in Indian cohorts^9,18^. Many variants identified in European cohorts are rare or absent in Indian populations^19^. For example, *LRRK2* p.G2019Ss, common in North Africans and Ashkenazi Jews, is infrequent in Indian PD patients^11,19^. Moreover, most PRS models are based on European datasets and may perform poorly in other populations due to differences in allele frequency, linkage disequilibrium (LD) and variant effect sizes^20^. This highlights the need for population-specific PRS development to improve risk prediction and enable more inclusive precision medicine approaches.

Existing Indian PD studies are limited by moderate sample sizes, a focus on known candidate genes, or reliance on SNP-array or exome-based approaches^21,22^. These methods primarily detect common coding variants and often miss structural variants (SVs), deep intronic variants, and regulatory changes that may contribute to disease risk. Recent discoveries highlight the role of such variant classes in PD pathogenesis. For instance, an intronic enhancer variant in *SNCA*^23^ and cryptic splice site variants in *PARK7*^24,25^ impact PD risk by affecting gene function.

In this study, we performed whole-genome sequencing (WGS) of 197 Indians with PD to map its genetic landscape in this underrepresented population. WGS offers a comprehensive platform for capturing the full spectrum of genomic variation across coding and non-coding regions, including rare single nucleotide variants (SNVs), SVs, repeat expansions, and splice-altering changes^26,27^. We aimed to uncover population-specific variation and novel loci, advancing understanding of PD genetic architecture in India.

## Results

### Cohort description

We enrolled 229 individuals in our cohort from the Movement Disorders Clinic at the National Institute of Mental Health and Neurosciences (NIMHANS), a tertiary referral hospital in India, including 197 PD patients from across India. 106 individuals had early-onset PD (EOPD), defined as age at onset (AAO) < 50 years, and 91 had late-onset PD (LOPD), with an AAO greater than 50 years. The average AAO was 38.95 ± 7.92 years for EOPD and 57.62 ± 5.54 years for LOPD. 72 out of 106 EOPD cases (67.9%), and 71 out of 91 LOPD cases (78%) were male. The dataset also included 32 healthy controls (HC). Supplementary Data S1 contains demographic information for all 229 samples. Principal component analysis of genetic data showed clustering of individuals by state (Supplementary Fig. S1B). The geographic distribution of these individuals is visualized in Supplementary Fig. S1A.

### Prioritization of protein-coding SNVs, splice variants and indels

We called genotypes for 229 samples at 29,932,165 variants using the GATK best practice variant calling pipeline^28^. We filtered out common variants with >1% minor allele frequency (MAF) in gnomAD v4^29^ global allele frequency, giving us 18,259,705 variants (61%). Among these, 131,141 (0.72%) were missense, frameshift, stop-gained or splice variants. 477 rare variants were in 69 genes previously implicated in PD (Supplementary Data S2). We removed variants found in more than one control, and those for which carrier genotypes didn’t match the known pattern of inheritance for the gene. These criteria filtered our list to 166 variants. We further prioritized these variants using American College of Medical Genetics and Genomics (ACMG) guidelines^30^.

**Fig. 1:**
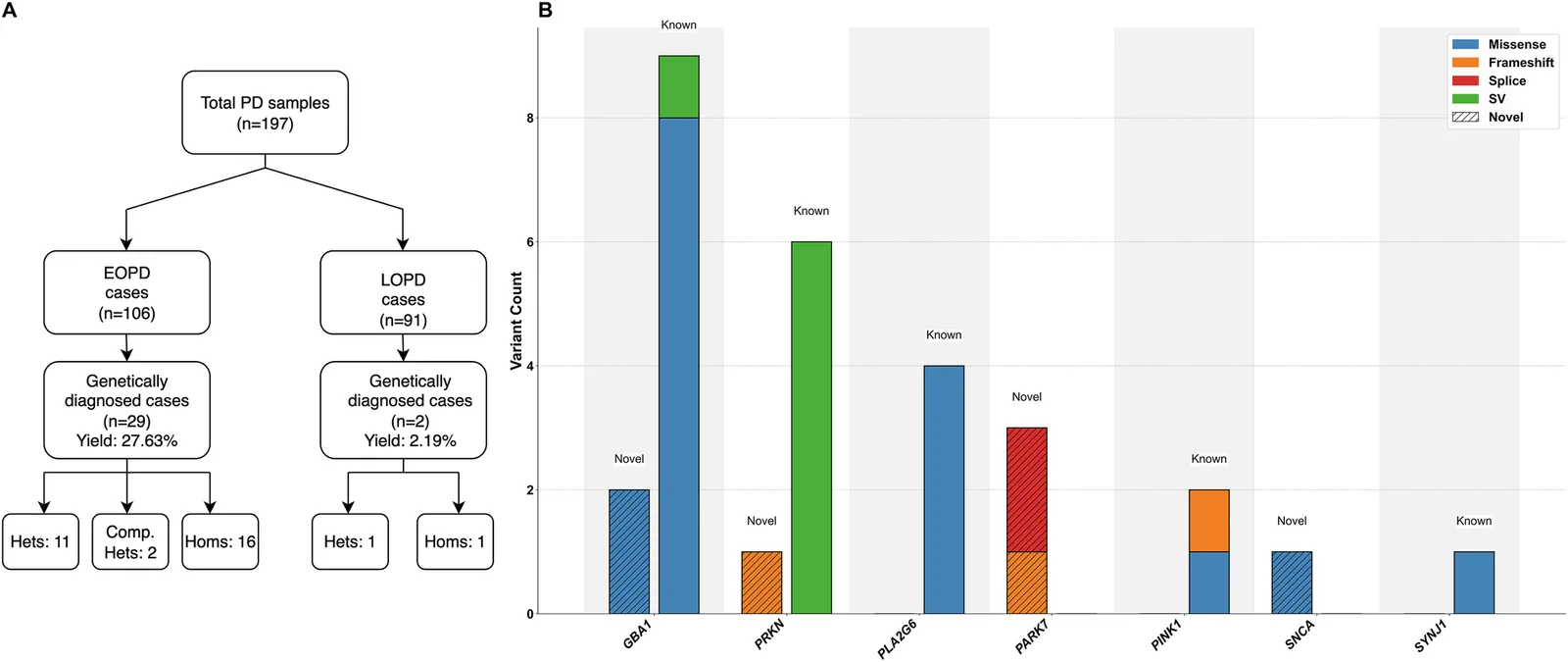
Genetic diagnostic yield and pathogenic variant distribution across PD-associated genes. **A** Illustrates the diagnostic yield, and the number of heterozygous (Hets), compound heterozygous (Comp. Hets), and homozygous (Homs) carriers in EOPD and LOPD cases. **B** Shows the distribution of pathogenic variants (*n* = 29 variants) across the analyzed PD-associated genes.

23 cases (Fig. 1A, B; Table. 1; Supplementary Data S3) carried pathogenic or likely-pathogenic (P/LP) variants in seven PD-associated genes: *GBA1, PRKN, PINK1, DJ1, SYNJ1, SNCA* and *PLA2G6*. Ten out of these 23 cases (all EOPD) were homozygous or compound heterozygous for the identified variants. For the two cases carrying compound heterozygous variants in *PLA2G6*, compound heterozygosity was confirmed experimentally by targeted ONT long-read amplicon sequencing. WhatsHap read-backed phasing of full-length amplicon reads (~5325 bp) spanning both variant positions simultaneously demonstrated that the two pathogenic variants reside on opposite alleles (trans configuration) in both cases, confirming true compound heterozygosity (Supplementary Material; Supplementary Table S3; Supplementary Fig. S10). The dominance of homozygous variants in EOPD is consistent with patterns of monogenic early-onset disease^31–33^. Population-level allele frequency comparisons for these variants are shown in Supplementary Fig. S2. Beyond the 69 established PD genes, we expanded our analysis to include 86 genes near 90 previously reported PD GWAS loci. We identified 2644 rare variants (MAF < 1%), and applied ACMG classification to identify two cases with known pathogenic, three with likely pathogenic, and 217 cases with variants of uncertain significance (VUS) (Supplementary Data S4).

**Table 1:** Pathogenic and likely pathogenic variants identified in 23 PD cases

Sample ID	Clinical phenotype	Variant ID	Protein change	Gene	Zygosity	Type	Age/AAO/Sex	State
YLOPD177	EOPD, Sporadic, PIGD, UPDRS part III (off): 59	c.90+1delG [novel]	p.?	*PARK7*	*hom*	splice	39/19/F	Karnataka
YLOPD185	EOPD, Familial, TD, UPDRS part III (off): 33	c.252+1G>T [novel]	p.?	*PARK7*	*hom*	splice	28/25/F	Karnataka
YLOPD-37	EOPD, Sporadic, TD, UPDRS part III (off): 38	c.456delT [novel]	p.Thr154fs	*PARK7*	*hom*	frameshift	30/25/M	Tamil Nadu
YLOPD-02	LOPD, Sporadic, PIGD, UPDRS part III (off): 48	c.776A>T [novel]	p.Tyr259Phe	*GBA1*	*het*	missense	66/60/M	Karnataka
YLOPD_96	EOPD, Familial, TD, UPDRS part III (off): 29	c.711G>C [novel]	p.Lys237Asn	*GBA1*	*het*	missense	57/47/M	Karnataka
YLOPD139	EOPD, Sporadic, TD, UPDRS part III (off):15	c.151G>A [novel]	p.Gly51Ser	*SNCA*	*het*	missense	54/47/F	Karnataka
YLOPD28	EOPD, Familial, TD, UPDRS part III (off): 29	c.374dupC [novel]	p.Asp190fs	*PRKN*	*hom*	frameshift	33/27/M	West Bengal
YLOPD211	EOPD, Sporadic, TD, UPDRS part III (off): 18	c.85_106delTACGGCTTGGGGCGGCCGGGCC [known]	p.Tyr29fs	*PINK1*	*hom*	frameshift	43/42/ M	Tamil Nadu
YLOPD157	EOPD, Sporadic, PIGD, UPDRS part III (off): 36	c.1153T>C [known]	p.Phe385Leu	*PINK1*	*hom*	missense	50/41/M	Tamil Nadu
YLOPD_62	EOPD, Sporadic, PIGD, UPDRS part III (off): 31	c.1448T>C [known]	p.Leu483Pro	*GBA1*	*het*	missense	44/38/M	West Bengal
YLOPD128	EOPD. Sporadic, PIGD, UPDRS part III (off): 59	c.1448T>G [known]	p.Leu483Arg	*GBA1*	*het*	missense	48/37/F	West Bengal
YLOPD29	EOPD, Sporadic, TD, UPDRS part III (off): 44	c.1448T>C [known]	p.Leu483Pro	*GBA1*	*het*	missense	30/26/M	Bihar
YLOPD184	EOPD, Sporadic, Indeterminate, UPDRS part III (off): 64	c.1448T>C [known]	p.Leu483Pro	*GBA1*	*het*	missense	56/45/F	Karnataka
YLOPD152	EOPD, Sporadic, PIGD, UPDRS part III (off): 37	c.1342G>C [known]	p.Asp448His	*GBA1*	*het*	missense	56/47/M	Karnataka
YLOPD193	EOPD, Sporadic, Indeterminate, UPDRS part III (off): 17	c.1342G>C [known]	p.Asp448His	*GBA1*	*het*	missense	51/48.5/M	Kerala
YLOPD227	EOPD Sporadic, Indeterminate, UPDRS part III (off): 45	c.827C>T [known]	p.Ser276Phe	*GBA1*	*het*	missense	35/31/M	West Bengal
YLOPD209	EOPD, Sporadic, TD, UPDRS part III (off): 55	c.667T>C [known]	p.Trp223Arg	*GBA1*	*het*	missense	43/39/M	Andhra Pradesh
YLOPD208	EOPD, Sporadic, Indeterminate, UPDRS part III (off): 37	c.509G>A [known]	p.Arg170His	*GBA1*	*hom*	missense	40/36/F	Odisha
YLOPD40	EOPD, Sporadic, PIGD, UPDRS part III (off): 29	c.492C>G [known]	p.Ser164Arg	*GBA1*	*het*	missense	46/41/M	West Bengal
YLOPD 20	EOPD, Sporadic, PIGD, UPDRS part III (off): 41	c.44T>C [known]	p.Leu15Ser	*GBA1*	*het*	missense	64/46/F	West Bengal
YLOPD_90	EOPD, Familial, Indeterminate, UPDRS part III (off): 60	c.773G>A [known]	p.Arg258Gln	*SYNJ1*	*hom*	missense	35/28.5/F	Karnataka
YLOPD238	EOPD, Sporadic, TD, UPDRS part III (off): 67	c.2341G>A; c.2239C>T [known]	p.Ala781Thr; p.Arg747Trp	*PLA2G6*	*compound het*	missense	50/37/F	Andhra Pradesh
YLOPD50	EOPD, Familial, TD, UPDRS part III (off): 77	c.2222G>A;c.1898C>T [known]	p.Arg741Gln; p.Ala633Val	*PLA2G6*	*compound het*	missense	23/21/M	Odisha

Each entry includes the sample id, clinical phenotype comprising disease subtype (EOPD: early-onset PD, LOPD: late-onset PD), family history (Familial, Sporadic), motor subtype (TD: tremor-dominant, PIGD: postural instability and gait difficulty), and motor severity assessed using the UPDRS Part III (off-state) score the variant ID (HGVS coding sequence nomenclature), protein change, the affected gene and zygosity status (het: heterozygous, hom: homozygous, compound het: compound heterozygous), type of mutation (missense, frameshift, or splice-site), current age, age at onset (AAO, in years), sex (M: male, F: female), and state of origin. Variants are classified as either *novel* (not previously reported in PD or reference databases) or *known* (previously described in PD cohorts or annotated in ClinVar/gnomAD).

Figure 1B shows the distribution of pathogenic variants (SNVs, indels, and SVs) found in PD-associated genes. *GBA1* was the most commonly mutated gene in our cohort, with 13 cases carrying P/LP variants. These included two variants previously not reported for PD pathogenesis, one of which was absent from gnomAD. This is unlike a few other genomic studies in Indian PD cohorts, which did not report a high number of *GBA1*^34^. 12 GBA1 variants were validated by ONT long-read amplicon sequencing (Supplementary material; Supplementary Fig. S8); one additional sample (YLOPD20), for which ONT sequencing failed due to DNA degradation, was confirmed by Sanger sequencing (Supplementary Fig. S9). Apart from the variants in these 23 diagnosed cases, we also screened for variants in 40 genes associated with parkinsonism, dystonia, and ataxia (Supplementary Data S1). Several additional variants were identified that may be relevant to disease etiology but currently lack sufficient evidence (in terms of zygosity or direct relevance to the phenotype) to be classified as causal. Two cases carried a heterozygous novel likely pathogenic *ATXN2* mutation. Eleven cases carried heterozygous pathogenic variants in genes primarily associated with recessive parkinsonism (*DNAJC6, VPS13C, SPG11* (two cases), *FBXO7, TH* (two cases), *CP, MYORG* (two cases), and *AOPEP)* (Supplementary Data S6). The list of all variants in 40 genes is listed in Supplementary Data S7.

We manually annotated and curated the list of Variant of Uncertain Significance (VUS) in the 69 PD-related genes to prioritize variants in this list (Supplementary Data S5), and to identify potential disease-relevant variants that were missed by previous annotation methods. One LOPD case carried a novel, highly damaging variant in *LRRK2* (pAla1442Pro), absent from gnomAD. While the VUS status for this variant is based on its unknown effect on protein structure, AlphaMissense^35^ predicts a strong effect on protein structure (pathogenicity score of 0.987), leading us to prioritize this as a likely causal variant that should be followed up in functional studies. Genes with rare VUS (Supplementary Data S8) seen in PD cases, were enriched in pathways of neurodegeneration (Supplementary Fig. S3A) (odds ratio 4.4; adjusted *P* = 1.26e−05), suggesting that this list of variants and genes includes truly pathogenic variants that are relevant for PD in this cohort. Genes with P/LP (Supplementary Data S9) variants were also enriched in PD (odds ratio 7.25; *P* = 2.9e−04) and lysosome pathways (odds ratio 14.53; *P* = 7.32e−06; Supplementary Fig. S3D, Supplementary Data S15).

### Pathogenic SVs in PD

We called SVs using five different SV callers, prioritizing those called by all five to minimize false positive calls. We specifically examined SVs overlapping exons of 69 PD-associated genes that are found in cases and no more than 1 control. After filtering for PD genes and inheritance pattern of the gene, no insertions or duplications were prioritized. Seven cases carried homozygous exon deletions in the *PRKN* gene, two of which are siblings which carry the same *PRKN* deletion, and one case carried a heterozygous *GBA1* variant (Supplementary Data S10; Fig. 1B). Visual inspection confirmed the presence of these deletions (Supplementary Fig. S7). The heterozygous *GBA1* structural variant was validated by ONT long-read amplicon sequencing (Supplementary material; Supplementary Fig. S8M).

Outside of the 69 PD-genes, one case also carried a heterozygous deletion in *CACNA1A*, a dystonia gene, removing exons 1 and 2 in a male with LOPD (Supplementary Data S11; Supplementary Fig. S7). Variants in *CACNA1A* have previously been associated with several neurological disorders, including episodic ataxia type 2, spinocerebellar ataxia type 6, and familial hemiplegic migraine type 1,^36,37^ pointing to its potential relevance for dystonia. The full list of identified deletions called by 5 callers is in Supplementary Data S12.

### PRS for PD are higher in cases

**Fig. 2:**
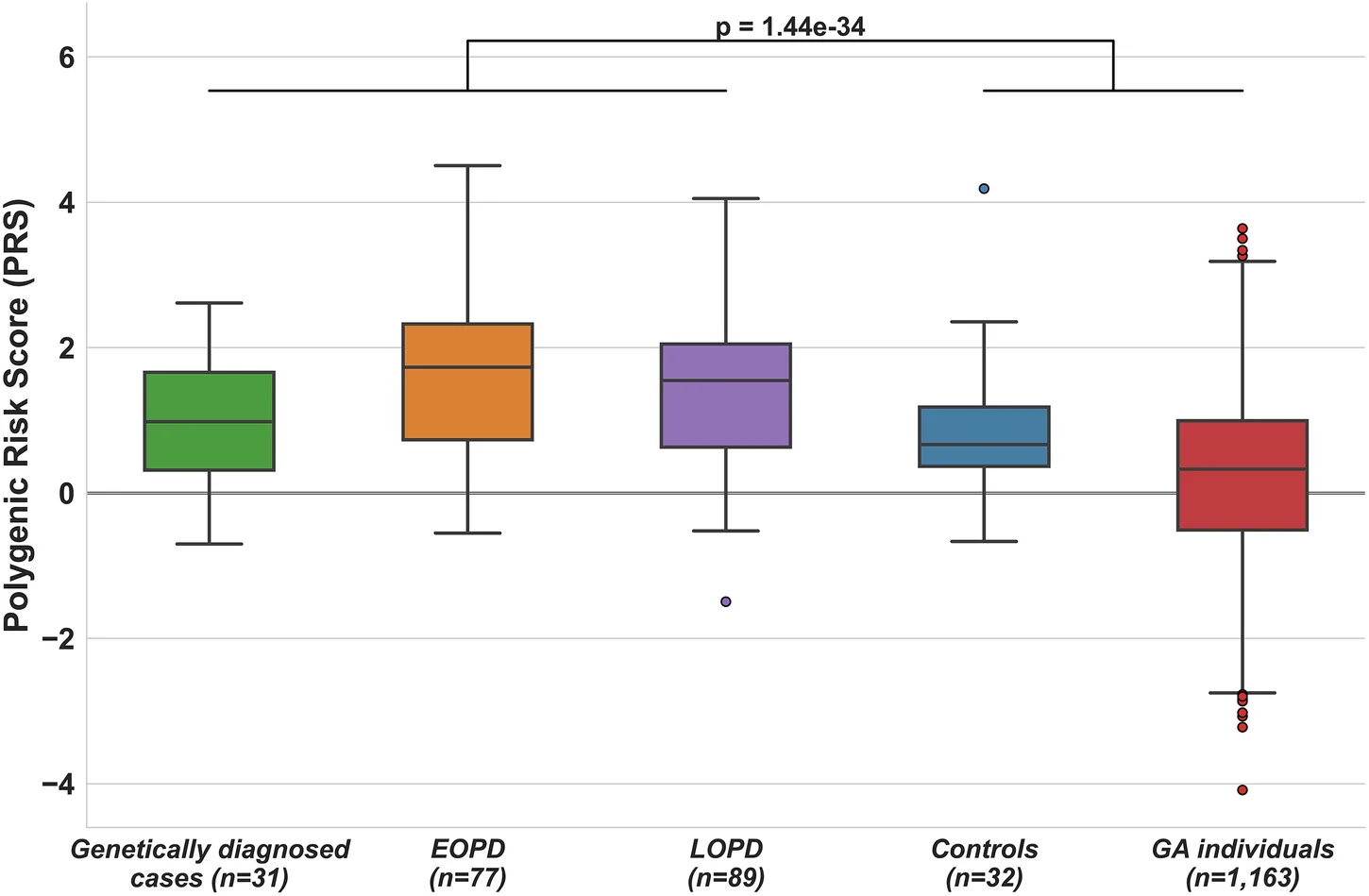
Distribution of PRS in cases, cases without a Mendelian variant, and controls. Distribution of Polygenic Risk Scores (PRS) values among five groups: genetically diagnosed cases, individuals with EOPD, individuals with LOPD), healthy controls (HC), and GenomeAsia individuals (GA).

PD cases have significantly higher PRS (Supplementary Data S13; Fig. 2 compared to both 32 internal controls and 1163 external controls from the GenomeAsia dataset^38^ (*P* < 1.44e−34; unchanged when run without siblings included, Supplementary Fig. S6). Our internal set of controls, drawn from healthy individuals from the same host site, show significantly non-zero PRS, pointing to higher background risks for PD from clinical settings.

Consistent with prior studies^39^, individuals with P/LP variants had qualitatively lower PRS relative to those without a genetic diagnosis, though this dataset is not adequately powered to identify significant differences. Cases carrying pathogenic variants are more likely to be driven by rare, high impact variants rather than a high burden of common risk variants.

### No gene with higher burden of functional variants in cases

PD cases carried significantly more P/LP variants (taken across the union of all genes) compared to controls (Fisher’s test *P* = 0.01), indicating that they carry a higher mutation load. To identify any potential genes enriched in burden among cases, we tested for rare variant gene-based burden in PD cases using gnomAD samples as controls. We used the consistent summary counts based rare variant burden test (CoCoRV) framework^31^, which incorporates systematic filtering, variant categorization, and ethnicity-stratified association testing while adjusting for multiple comparisons. Although a few genes showed nominal significance (Supplementary Data S14), none remained significant after false discovery rate (FDR) correction (Supplementary Fig. S4; unchanged when run without siblings included, Supplementary Fig. S5). These findings suggest that the current cohort lacks sufficient statistical power to detect gene-level associations, underscoring the need for larger sample sizes to identify novel candidate genes in PD progression.

## Discussion

In this whole genome-based study of genetic architecture of PD from India, we report a diagnostic yield of 15.73%, with a higher yield in EOPD (27.63%) than LOPD (2.19%). A monogenic cause could be attributed in 50% of familial cases compared to 12.36% of apparently sporadic cases. Pathogenic and likely pathogenic variants were identified in *GBA1 (*45.16% of all identified pathogenic variants)*, DJ1* (9.67%)*, PINK1* (6.45%)*, PLA2G6* (6.45%)*, PRKN* (25.80%)*, SNCA* (3.22%), and *SYNJ1* (3.22%).

*GBA1* variants are important genetic risk factors for PD^40^. Across different populations, the frequency of *GBA1* variants in PD ranges from 3% to 20%^9,41,42^. Rare variants in *GBA1* are present in 5–15% of the Indian population^21^, but many remain unconfirmed as causative for PD^43^. While this may reflect varying importance of the *GBA1* gene in different Indian subpopulations, the choice of reference genome used in analysis may also contribute to under-reporting of *GBA1* variants^44^. The analysis of *GBA1* using short-read sequencing is technically challenging due to its high sequence homology with the nearby pseudogene *GBAP1*, which can lead to read misalignment and inaccurate variant calling. These limitations complicate variant interpretation and may affect the reliability of *GBA1* variant detection in short-read datasets. Long-read ONT amplicon sequencing was performed to validate the variant calls, confirming variants with high confidence. Importantly, ONT sequencing corrected the ALT allele assignment for three samples at position chr1:155,235,252 variants miscalled by short-read WGS potentially due to misalignment with the *GBAP1* pseudogene, underscoring the importance of long-read sequencing for accurate *GBA1* variant characterization.

In our cohort, *GBA1* alterations were detected in 7.10% of patients. Only a single individual reported a second-degree relative with PD, and all other cases appeared sporadic. As with previous observations^21,40,42^, heterozygous pathogenic *GBA1* variants in our cohort were associated with an earlier age of onset, with 13 of 14 *GBA1* cases presenting with EOPD. The relatively high frequency of these variants in our sample is clinically relevant, as they have been linked to more rapid progression of motor dysfunction and greater cognitive decline^45^.

Three previously undescribed variants in the *PARK7* (*DJ1*) gene were discovered in this study. Two are homozygous splice-site changes identified in individuals with familial PD. The third is a homozygous frameshift variant predicted to be likely pathogenic. In the global literature, *PARK7* variants are rare, with limited clinical data available on their contribution to early-onset forms of PD^46–48^. Biallelic pathogenic variants in *DJ1* are estimated to account for around 1% of EOPD, frequently associated with diverse or atypical clinical presentations. Consistent with previous reports, all cases identified in our cohort had EOPD, with symptom onset ages of 19, 25, and 38 years.

One case with EOPD (AAO of 21 years) had a novel heterozygous missense variant in the *SNCA* gene. *SNCA* variants are rare in Indian populations and heterozygous missense changes are an uncommon cause of parkinsonism^49,50^. Previous studies have demonstrated that G51D mutation of the *SNCA* gene leads to reduced synaptic localisation of α-synuclein, proteomic evidence of early synaptic dysfunction, and an increased propensity for α-synuclein pathology^51^. The novel *SNCA* p.Gly51Ser variant identified in this cohort occurs at the same mutational hotspot as the pathogenic p.Gly51Asp variant and is absent from gnomAD and is classified as LP by ACMG guidelines, but further functional investigation is warranted to clarify its biological and clinical implications. Pathogenic biallelic variants in the *PRKN* gene contribute to ~10–20% of EOPD cases globally^52^, although a considerable number of patients are found to carry VUS. Within our study population, eight cases had *PRKN* variants. Of these, one carried a previously undescribed homozygous frameshift mutation, while the other seven had SVs. Seven affected individuals had EOPD, with disease onset ranging from 22 to 28 years, whereas one patient had LOPD. Prior studies indicate ~62% of *PARK‑PRKN* (PARK2) cases manifest as EOPD, with ~16% presenting as juvenile‑onset PD^53^. Exon 3 is affected in five out of seven patients with *PRKN* exon deletions. Exons 3–8 of *PRKN* span the central core of FRA6E, a well-established mutation hotspot and the likely mechanistic basis for the clustering of SVs in this region^54^.

Two EOPD patients carry known biallelic pathogenic variants in *PINK1*, one with a missense mutation and the other with a frameshift mutation. Two additional cases carry compound heterozygous variants in *PLA2G6*; one case carries (*p*.Ala781Thr and *p*.Arg747Trp), the latter being a known pathogenic variant supported by functional studies. Another case carried (p.Arg741Gln and p.Ala633Val), both of which are pathogenic. Another PD case carried a known homozygous missense variant in *SYNJ1*, with symptom onset at 31 years. We also identified two cases with a novel variations in *ATXN2* gene which warrants further exploration. Intermediate range of repeats and other variations in this gene are known to present with PD-like features and influence the phenotype in other neurodegenerative disorders. Large cohort studies and meta-analyses further highlight ethnic variability and pleiotropic effects, linking *ATXN2* to PD, ALS, and SCA2^55–57^. This suggests that *ATXN2* may play a broader role in neurodegeneration, with potential relevance for parkinsonism. Notably, the individuals in our cohort carry heterozygous point variants rather than the more common repeat expansions. While these results align with the emerging view of *ATXN2* as a broad contributor to neurodegeneration, we present these novel variants as candidates for further investigation. Future functional studies will be essential to definitively establish their pathogenicity and to determine if point mutations in *ATXN2* confer a similar risk profile to that of established repeat expansions. We also identified many VUS in PD genes; among them one *LRRK2* variant (p.Ala1442Pro) was found to be highly damaging and predicted to be pathogenic by AlphaMissense. This variant has previously been reported in an Australian familial PD case as a novel mutation associated with levodopa-responsive parkinsonism^58^, and more recently, functional studies demonstrated that p.Ala1442Pro increases *LRRK2* kinase activity and alters microtubule binding^59^, supporting its pathogenic potential. We also evaluated previously reported protective *LRRK2* haplotypes (N551K-R1398H-K1423K)^60^. Neither PD cases nor healthy controls in our cohort carried these protective variants, and consequently, no enrichment could be detected in controls relative to cases. Given the limited sample size, this absence could be due to limited statistical power rather than a true absence of protective haplotypes in the population. Larger cohorts will be required to reliably assess the frequency and protective effect of *LRRK2* haplotypes in Indian populations. The genes with VUS in PD cases are enriched for neurodegeneration pathways; this strongly suggests that the VUS set includes many likely causal variants that require more functional evidence and better variant prioritization. The identification of rare variants in our cohort underscores the need for comprehensive genetic analysis and careful phenotyping to improve our understanding of genotype-phenotype relationships and enhance early diagnosis, risk assessment, and tailored management strategies for PD patients in diverse populations.

Our results demonstrate that European-derived PRS maintain predictive value in Indian populations. This suggests that the overall polygenic contribution to PD risk is conserved across populations despite differences in individual risk variants and allele frequencies. Controls drawn from the same clinical center (one focused on mental health and neuroscience) show significantly higher PRS than external controls, perhaps pointing to the shared genetic architecture between neurological, psychiatric, and neurodegenerative phenotypes. This also points to potentially higher PD risk for carriers of other brain-related disorders^61^.

The Indian population remains under-represented in PD genetic research, despite the high burden of the disease. Compared to European cohorts, where population homogeneity and easier patient recruitment have facilitated large-scale studies, insights into PD genetics in India are still limited. Given that populations differ in allele frequencies, LD patterns, and effect estimates, no single population can fully elucidate the genetic underpinnings of PD. This underscores the scientific and clinical imperative to expand genetic research in diverse populations such as India, to capture population-specific risk variants and enhance understanding of the global genetic architecture of PD. Genes with likely pathogenic variants in cases are enriched in lysosomal pathways, underscoring the relevance of PD etiology^62^.

Finally, while our current study is underpowered to detect any genome-wide significant associations, this cohort provides a valuable foundation for larger collaborative studies that could identify novel PD loci specific to Indian populations. We also demonstrate the clinical utility of comprehensive genetic screening in Indian PD patients and highlight the need for population-specific diagnostic approaches.

## Methods

### Participant selection criteria

Diagnoses of PD was based on the International Parkinson and Movement Disorders Society (MDS) clinical diagnostic criteria for PD^63^ applied by the trained neurologists (PKP, RY, RM, NK, PM, and VVH). Between 2022 and 2024, all patients diagnosed with PD during routine clinical evaluation were offered participation in this study. Demographic and clinical details were collected during a structured interview which also included details of family history of PD, AAO, and clinical details such as motor and non-motor symptoms. A small number of HC were carefully age- and sex-matched to PD cases and recruited from the same geographic and ethnic background to minimize population stratification. Individuals with a personal or family history of PD or other neurodegenerative conditions were excluded from the HC group.

### Ethical considerations

The study was conducted in accordance with the Declaration of Helsinki. Institutional Ethics Committee approval from NIMHANS and the Centre for Brain Research was obtained prior to the study (ethics approval number CBR/42/IEC/2022). All participants provided written informed consent, acknowledging voluntary participation, and the use of their anonymized genetic data for research purposes.

### Genomic DNA processing

We collected whole blood from the study participants and extracted genomic DNA using standard protocols (QIAamp DNA Blood Midi Kit, Qiagen). We measured DNA concentration using the Qubit fluorometer (Thermo Fisher Scientific, USA). We constructed DNA libraries using TruSeq DNA PCR-free kit from Illumina. Samples were sequenced on the Illumina NovaSeq 6000 using 150 bp paired-end reads, achieving an average coverage of 54X. We converted raw BCL files to FASTQ format using bcl2fastq2 v2.20 (Illumina, Inc.). We assessed read quality using FastQC v0.12.1 and MultiQC v1.31^64^, and trimmed adapters and low-quality bases using Trimmomatic v0.39^65^.

### Variant calling and interpretation

We aligned sequencing reads to the GRCh38 reference genome without alt contigs using BWA-MEM2 v2.2.1^66^. We followed Genome Analysis Toolkit (GATK) v4.4.0 best practices^28^ for variant calling and preprocessing, including duplicate marking, base quality score recalibration, joint genotyping, and variant quality filtering. We annotated variants using SnpEff^67^ and ANNOVAR^68^ with reference databases including gnomAD genome database. For splice variants, we used SpliceAI^69^ score >0.8 to filter potential splice disrupting splice variants. We defined rare variants as those with a MAF < 1% in gnomAD v4 global allele frequency to classify them as P, LP, VUS, or benign as per ACMG guidelines.

We systematically evaluated 69 established PD-associated genes and 40 other genes associated with other movement disorders with parkinsonism (Supplementary Data S2) for rare pathogenic and candidate variants. We performed ONT long-read amplicon sequencing on all samples carrying *GBA1* variants to validate variant calls and avoid misalignment with the highly homologous *GBAP1* pseudogene. ONT long-read amplicon sequencing was also performed for *PLA2G6* to resolve the phase of compound heterozygous variants. Amplicon primers for *GBA1* and *PLA2G6* are provided in Supplementary Table S1. Primers used for Sanger confirmation are provided in Supplementary Table S2.

### SVs calling and prioritization

We called SVs using five tools: CNVnator^70^ (v0.4.1), LUMPY^71^ (v0.3.1), Manta^72^ (v1.6.0), Delly^73^ (v1.2.6), and BreakDancer^74^ (v1.4.5). For insertions, we used Manta, BreakDancer, INSurVeyor^75^ (v1.1.3) and Delly, since CNVnator and LUMPY don’t call insertions. We analyzed deletions, insertions, duplications, and inversions considering only variants with FILTER = PASS. VCFs for each sample were merged using SURVIVOR^76^ (v1.0.3) with the following parameters: maximum distance between breakpoints = 1000 bp, minimum support = 1 caller, require same SV type and strand, enable distance estimation, and a minimum SV size of 50 bp. We prioritized high-confidence variants supported by all callers (SUPP = 4 for insertions, and SUPP = 5 for all other classes) that overlapped exons in any of the 69 PD-associated genes. Final candidate variants were assessed for pathogenicity according to ACMG criteria and visually validated using the Integrative Genomics Viewer (IGV, v2.16.2)^77^.

### Polygenic risk score (PRS)

We estimated PRS for our dataset using LDpred-2 v1.11.6^78^. We used summary statistics from the largest European-ancestry PD GWAS as the base dataset^10^. To ensure high-quality variant data, SNPs with a genotype missing rate >1% were excluded along with individuals with >1% missing genotypes. We filtered out SNPs that significantly deviated from Hardy-Weinberg equilibrium (*p* < 1 × 10⁻⁶) and with MAF < 1%. To reduce redundancy due to LD, we performed LD pruning using a sliding window of 200 SNPs, moving 50 SNPs at a time, and retained variants with pairwise r² values below 0.25^79^. Common SNPs filtered from the two datasets were restricted to those present in HapMap3+ reference panel to ensure high variant inclusion. Finally, we computed LD matrix which along with the base data was used as input for the LDpred2-auto model^78^. Since our dataset has very few controls, we used 1163 samples from the GenomeAsia project^38^ to assess PRS in an external ancestry-matched control population. These controls were recruited from outpatient clinics and population-based studies across multiple cities in India, and were genotyped using the Illumina Global Screening Array, which includes over 600,000 genome-wide markers^80^. The distributions of PRS were compared between cases and controls as well as among PD subgroups categorized based on the presence of pathogenic variants.

### Pathway enrichment analysis

We performed geneset enrichment analysis to investigate the biological relevance of rare variants in PD cases. We used Enrichr^81^ to test enrichment against the KEGG_2021_Human, Reactome, and Gene Ontology (GO) Biological Process databases. Pathways with an adjusted *P*-value < 0.05 (Benjamini–Hochberg false discovery rate) were considered significantly enriched.

### Gene burden analysis

To identify genes with an excess burden of rare variants associated with PD, we used the CoCoRV framework. We compared our case cohort against gnomAD v4 exome coverage summary statistics, filtering DP ≥ 10, GQ ≥ 20 and excluding genomic regions flagged as problematic in gnomAD (segmental duplications, low-complexity regions, and other blacklisted sites based on gnomAD QC). To account for ancestry differences between cases and controls, cases were assigned to gnomAD v4 genetic ancestry groups using a gnomAD’s pre-trained random forest classifier. We limited analysis to regions with ≥10X coverage in ≥90% of samples and only retained loss-of-function and missense variants with REVEL ≥ 0.65. Burden testing was performed using ancestry-matched gnomAD exome South Asian (SAS) summary data under a dominant genetic model with MAF ≤1e−2, while accounting for LD. False discovery rate was controlled using a resampling-based approach, and potential residual confounding was evaluated using genomic inflation factor (λ).

## Supplementary information

Supplementary material

Supplementary Data

## Data Availability

The datasets generated in this study are available from the corresponding author on reasonable request.
