# Supplementary material for: Whole-genome sequencing of 197 cases with Parkinson’s disease reveals novel pathogenic variants in the Indian population
Source: NPJ Parkinsons Dis. 2026 Jul 6;12:264. doi: 10.1038/s41531-026-01462-0 (PMC13720001; doi:10.1038/s41531-026-01462-0)
Supplement: Supplementary file 1 [file 41531_2026_1462_MOESM1_ESM.pdf]

Whole-genome sequencing of 197 cases with Parkinson’s Disease reveals novel pathogenic variants in the Indian population

Supplementary Material

Contents:

1. Supplementary Figures

2. IGV snapshots of SV deletions

3. ONT Long-Read Amplicon Sequencing: Validation of *GBA1* and *PLA2G6* Variants

4. IGV snapshots for GBA1 validation - using long read ONT

5. IGV snapshots of haplotagged BAM files:

6. Burden testing, and PRS results

7. Supplementary Data column descriptions

8. Legends of Supplementary Data

Supplementary Figures

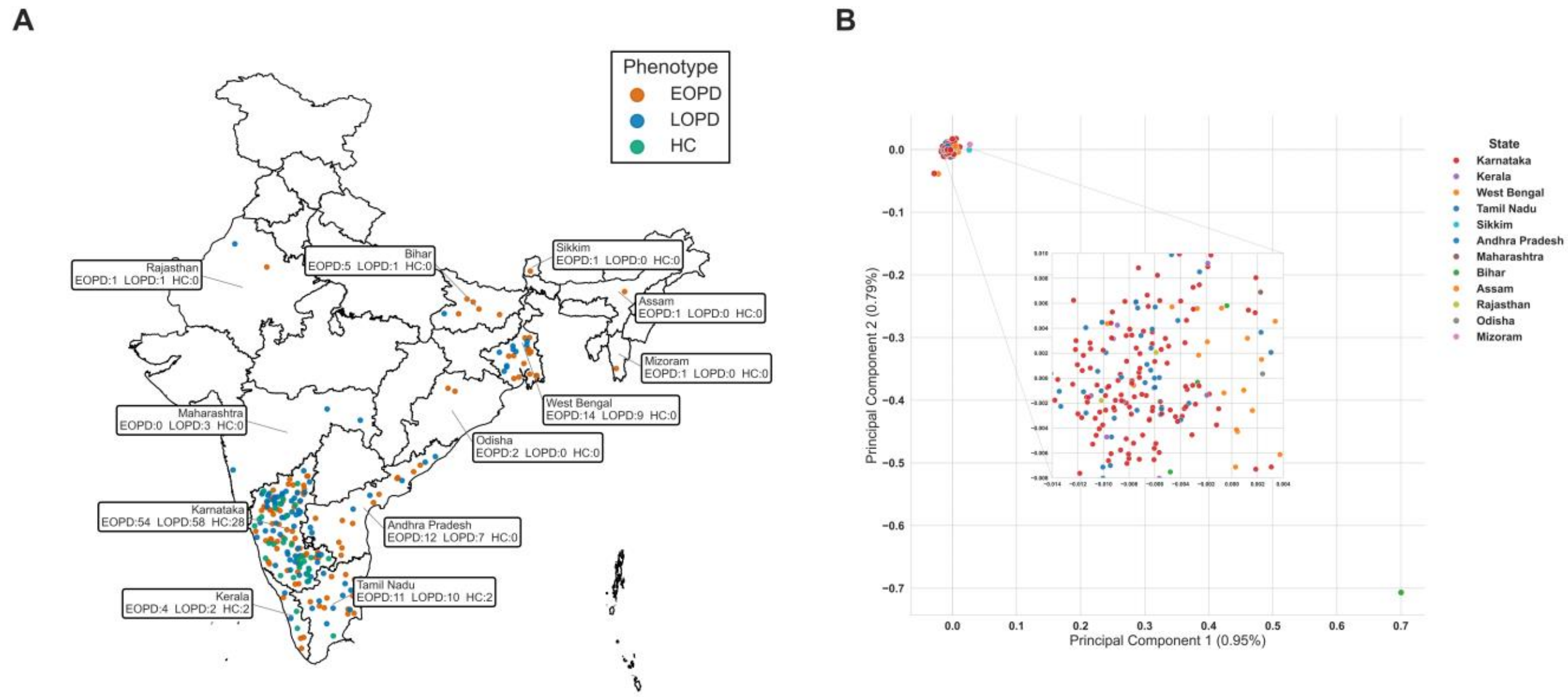

Figure S1. Geographic distribution and PCA plot of SNP data of the SKAN cohort. **(A)** Map of India showing the geographic origin of study participants, colored by phenotype (EOPD = early-onset PD; LOPD = late-onset PD; HC = healthy controls). State-level sample counts are indicated. **(B)** Principal component analysis (PCA) of SNP genotype data from all unrelated individuals. The plot shows PC1 vs PC2, which explain 0.95% and 0.79% of the total genetic variance, respectively. Each point represents an individual, colored by their state. The clustering along PC1 or PC2 reflects major sources of genetic structure in the dataset, potentially due to population stratification, geographic origin, or phenotypic differentiation.

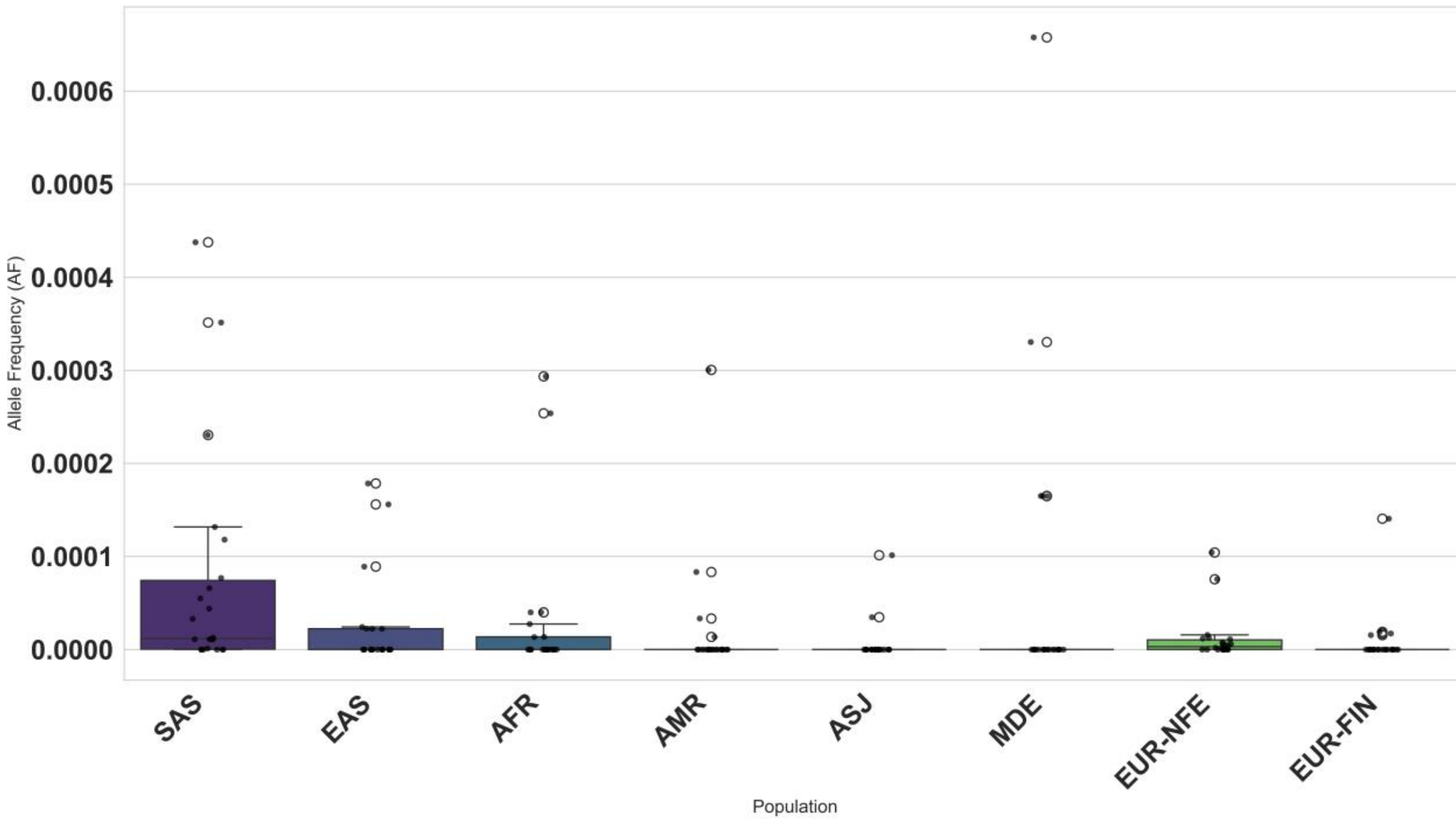

Figure S2. Mean Allele Frequencies of Pathogenic Variants in PD Genes Across Populations. Bar plot showing the mean allele frequency (AF) of pathogenic variants identified in known Parkinson's disease (PD) genes, stratified by population. The Y-axis indicates the mean AF, and the X-axis shows the population groups. Each bar represents the average frequency of pathogenic alleles within that group. This comparison highlights potential population-level differences in the burden of disease-associated variants.

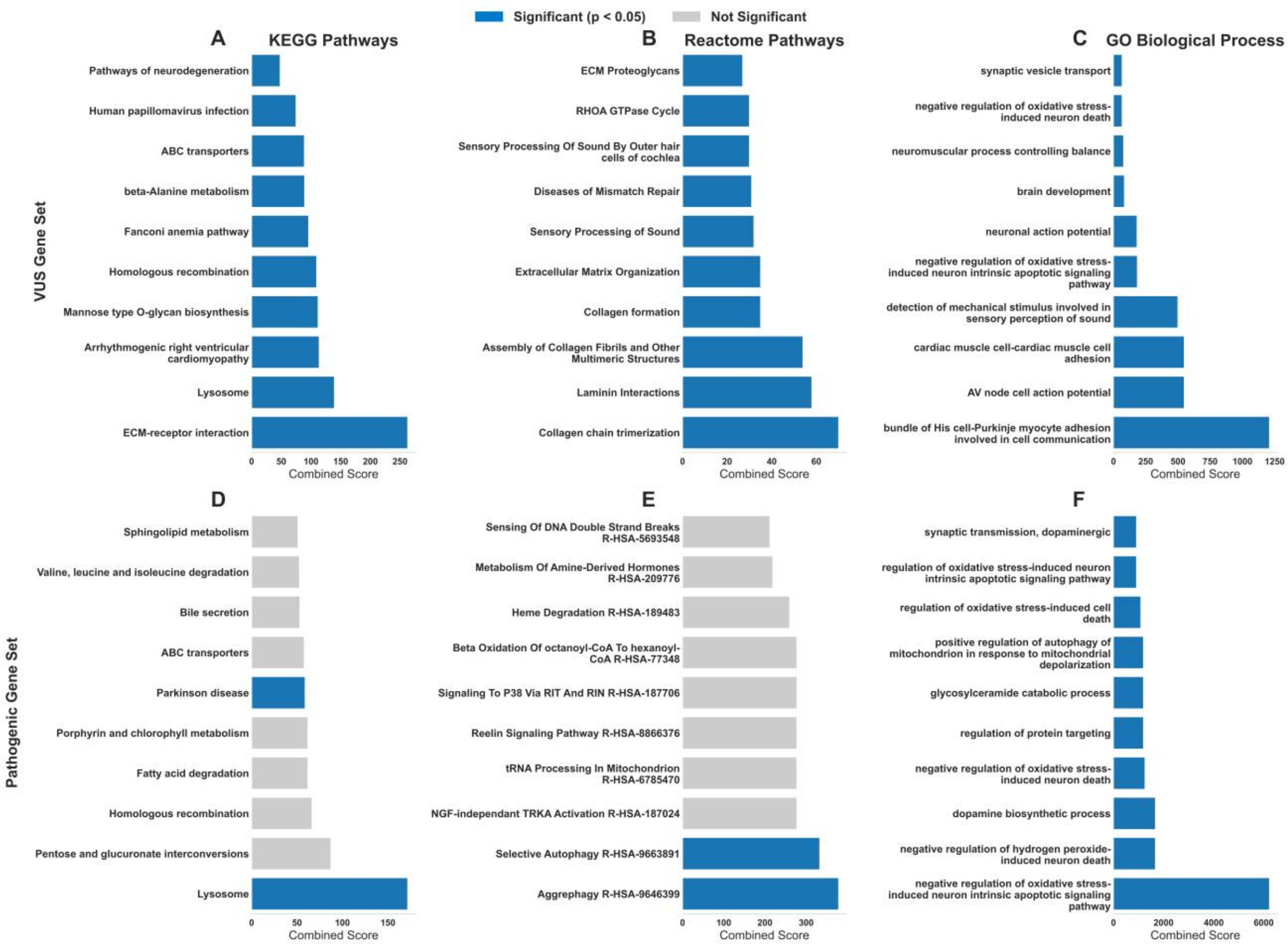

Figure S3. Pathway enrichment analysis of genes with variants of uncertain significance (VUS) and pathogenic/likely pathogenic variants. Top enriched pathways for the VUS gene set (top row, A-C) and the pathogenic gene set (bottom row, D-F). Analyses were performed using three databases: KEGG (A, D), Reactome (B, E), and GO Biological Process (C, F).

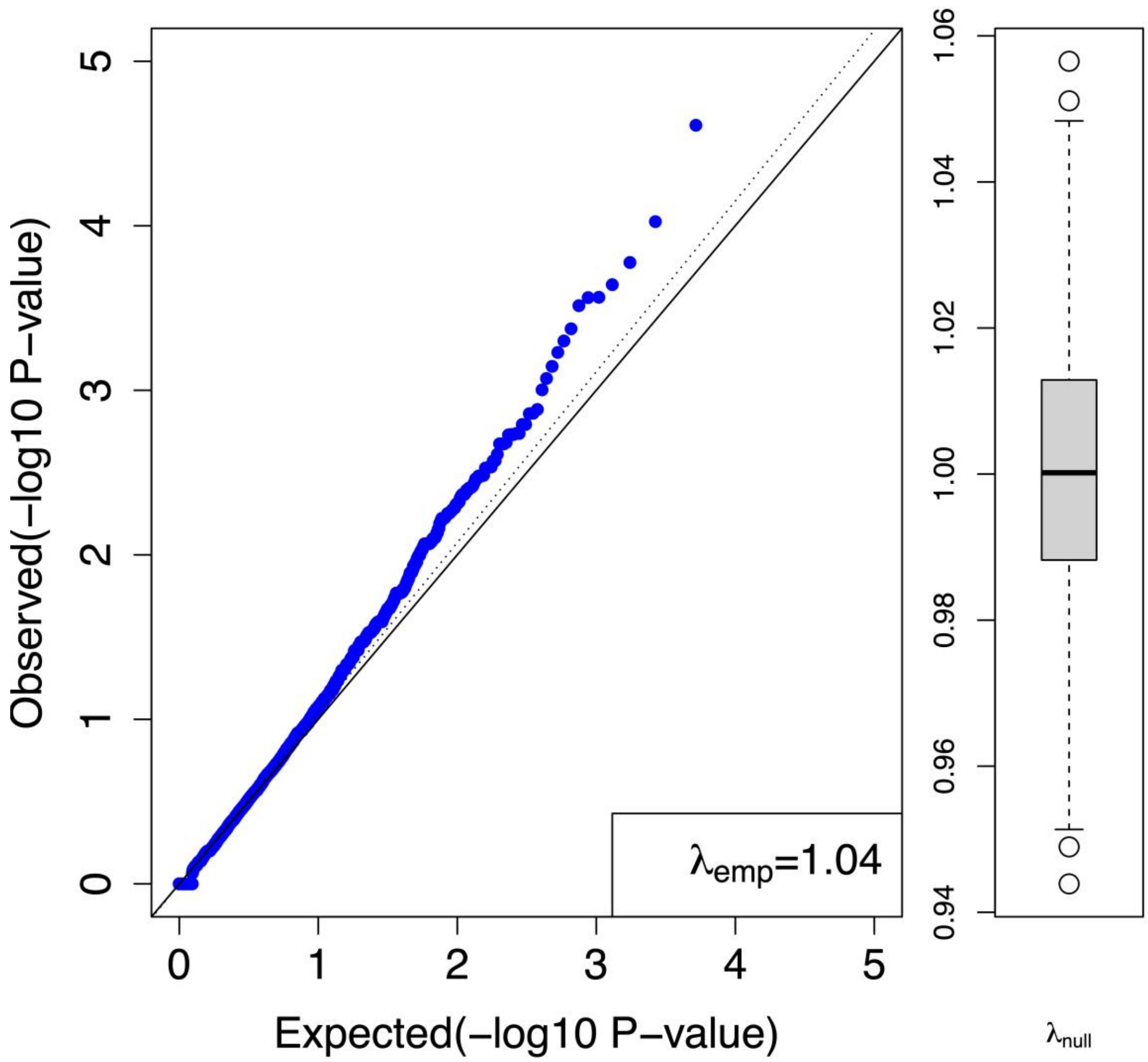

Figure S4. ***Quantile-Quantile (QQ) Plot of gene burden analyses in PD.*** QQ plot illustrating the distribution of observed versus expected p-values from gene-based rare variant burden testing using the CoCoRV framework ($\lambda = 1.04$). The slight deviation from the null suggests potential signals, but the modest inflation factor and limited number of significant hits indicate that the analysis remains underpowered due to the small sample size (n = 197 PD cases).

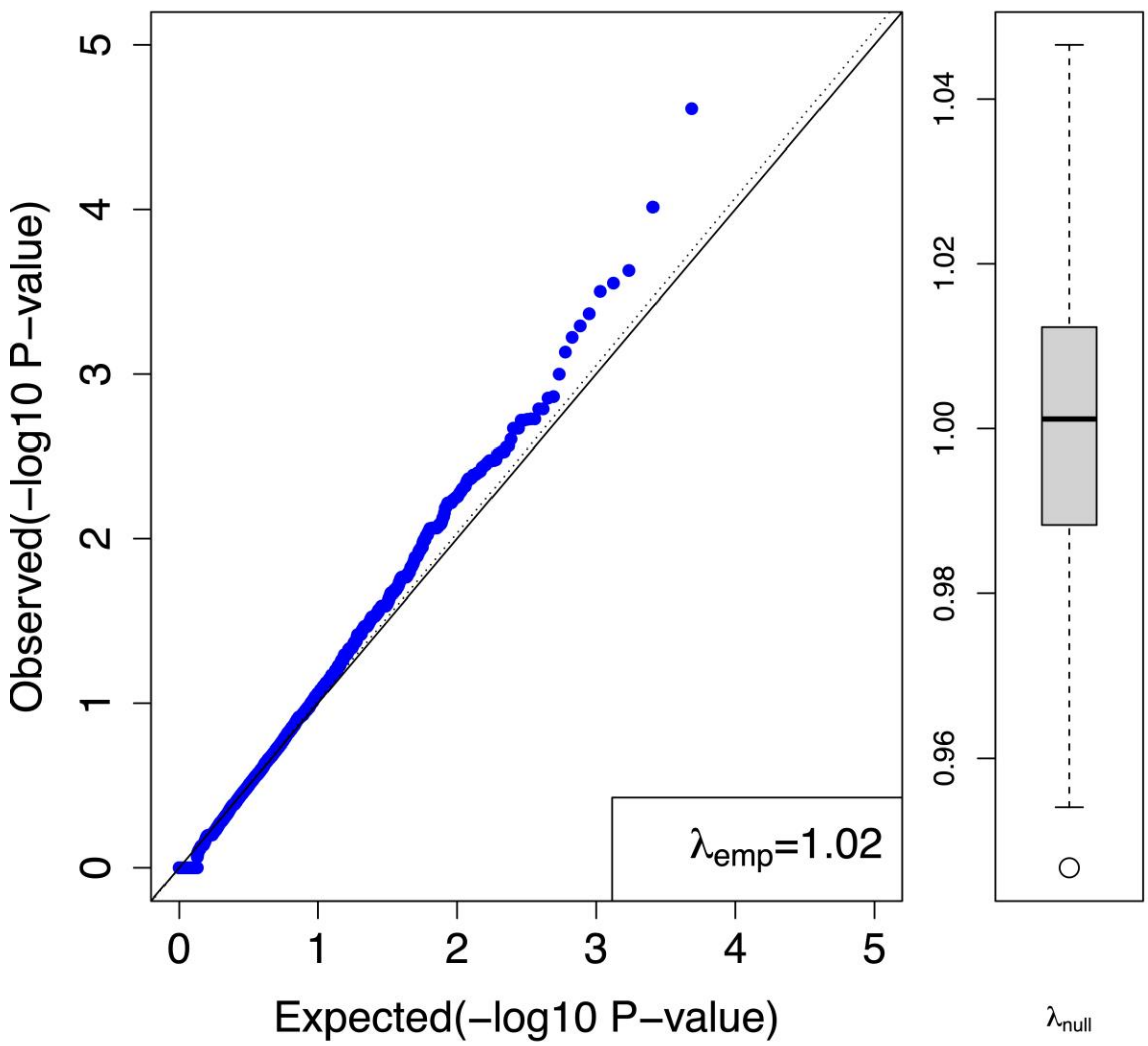

Figure S5. Quantile-Quantile (QQ) plot of gene burden analyses following exclusion of related individuals. The plot displays the distribution of observed versus expected p-values from gene-based rare variant burden testing using the CoCoRV framework, following exclusion of related individuals (YLOPD21 and YLOPD21e). The genomic inflation factor λemp = 1.02 falls within the empirical null distribution, confirming no inflation and consistency with the primary analysis.

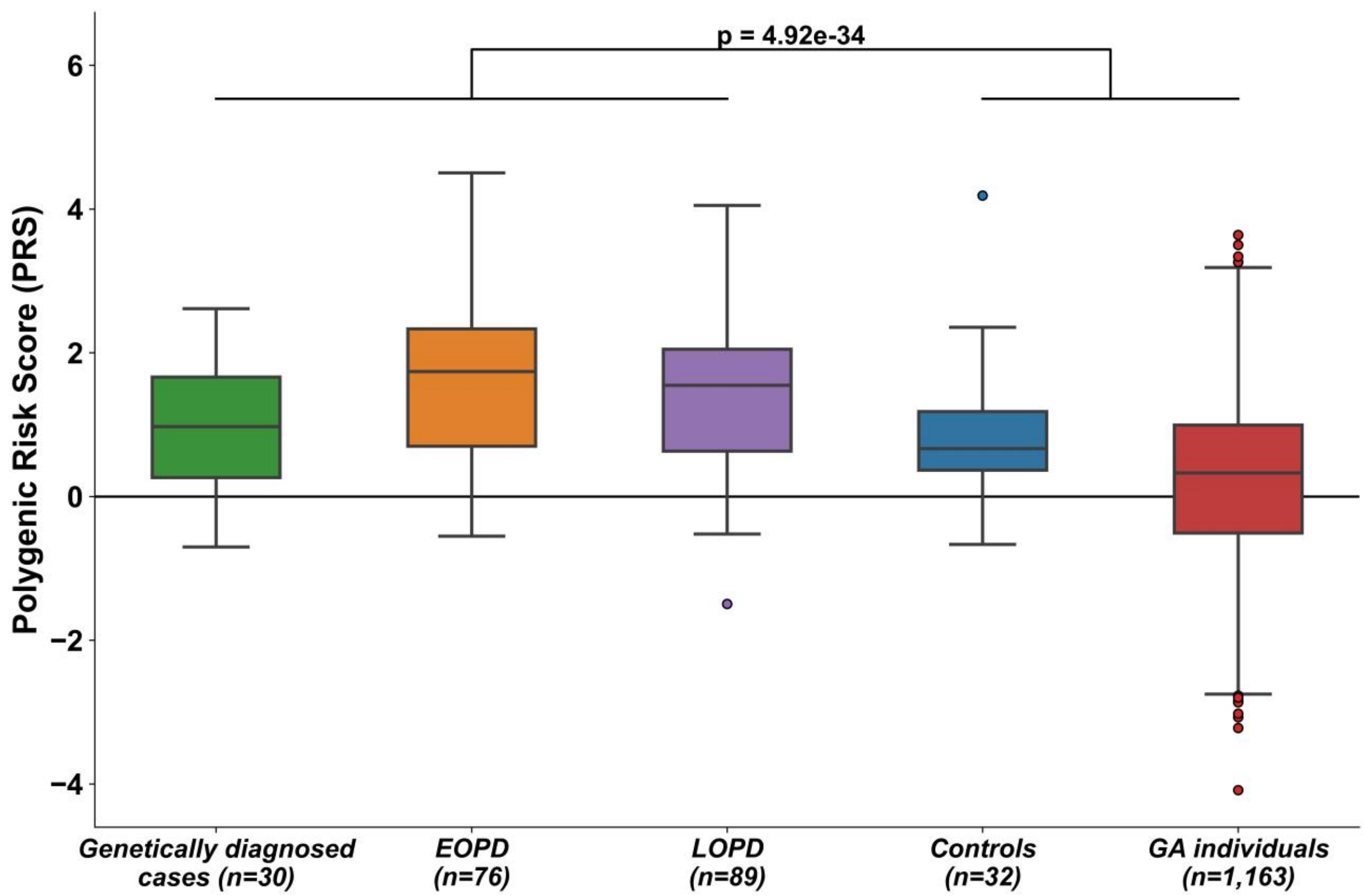

Figure S6. Polygenic Risk Score (PRS) distribution across case and control groups following exclusion of related individuals. PRS remains significantly elevated in PD cases relative to controls (P < 4.92e-34) following exclusion of related individuals (YLOPD21 and YLOPD21e), consistent with the primary analysis.

IGV Snapshots of SV deletions

All seven homozygous *PRKN* deletions reported in this study were called by five independent SV callers and manually inspected in IGV (Figure S7). Upon manual inspection in IGV, each deletion showed three key features expected of a true homozygous SV: a complete loss of read coverage across the deleted region, an absence of reads spanning the breakpoints, and clearly defined deletion boundaries. While this multi-caller consensus approach represents a rigorous and widely accepted standard for high-confidence SV calling, we acknowledge that orthogonal wet-lab validation, such as Multiplex Ligation-dependent Probe Amplification (MLPA) was not performed.

A)

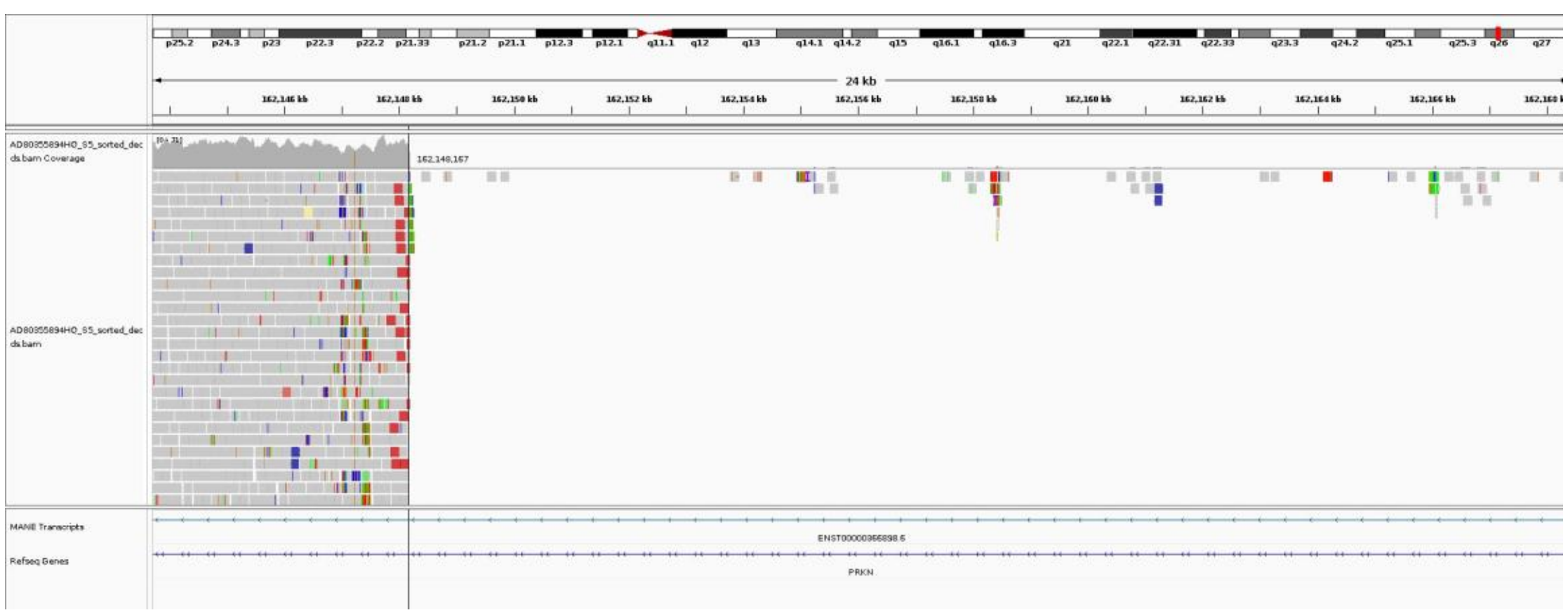

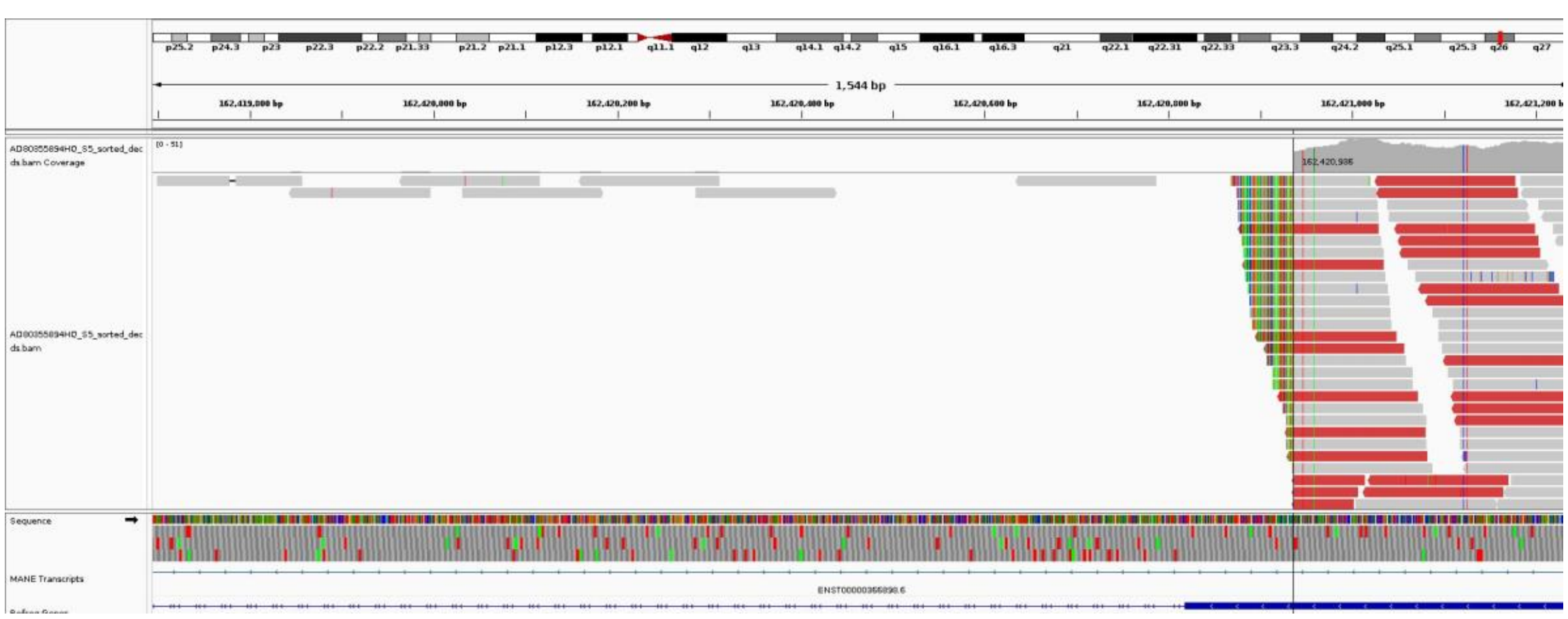

B)

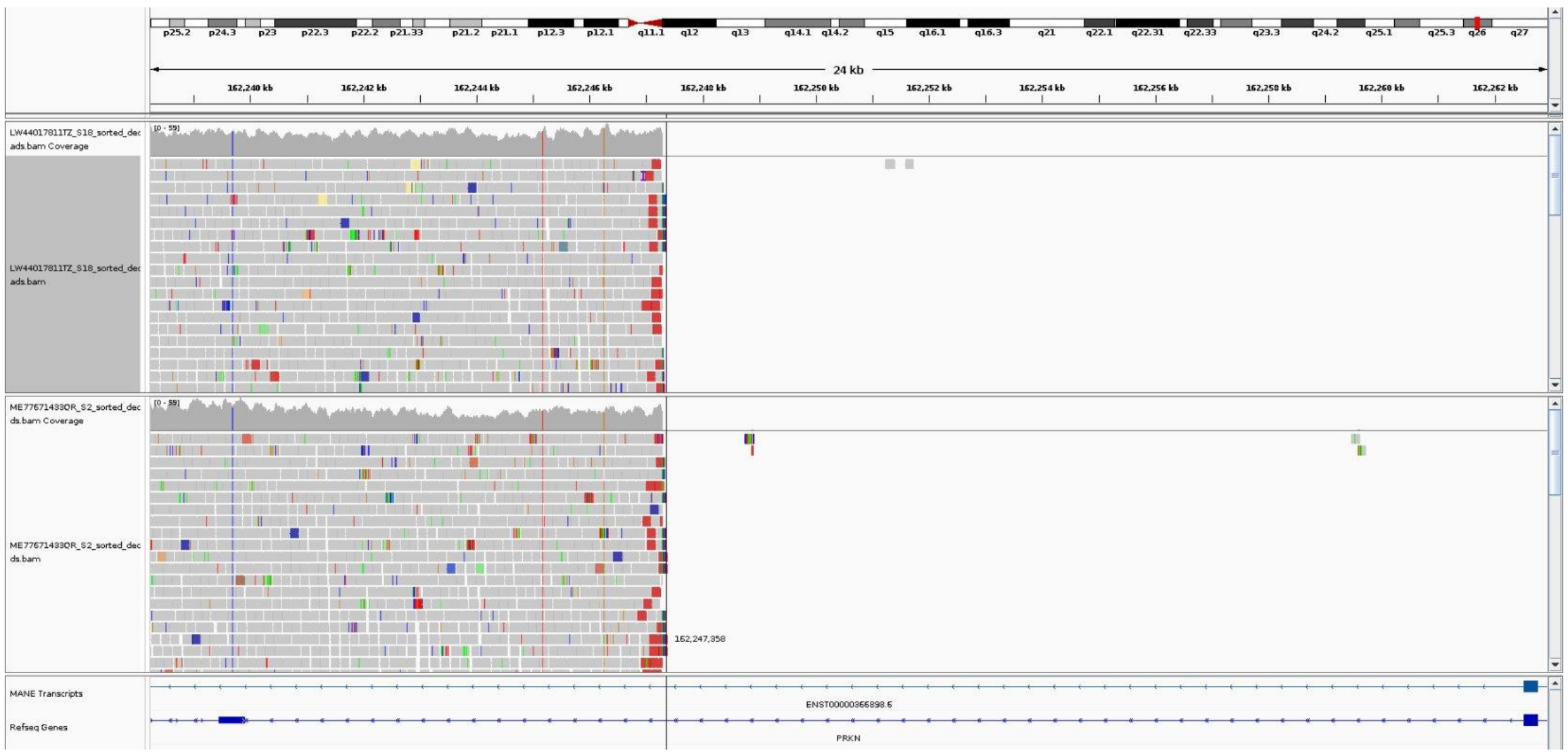

C)

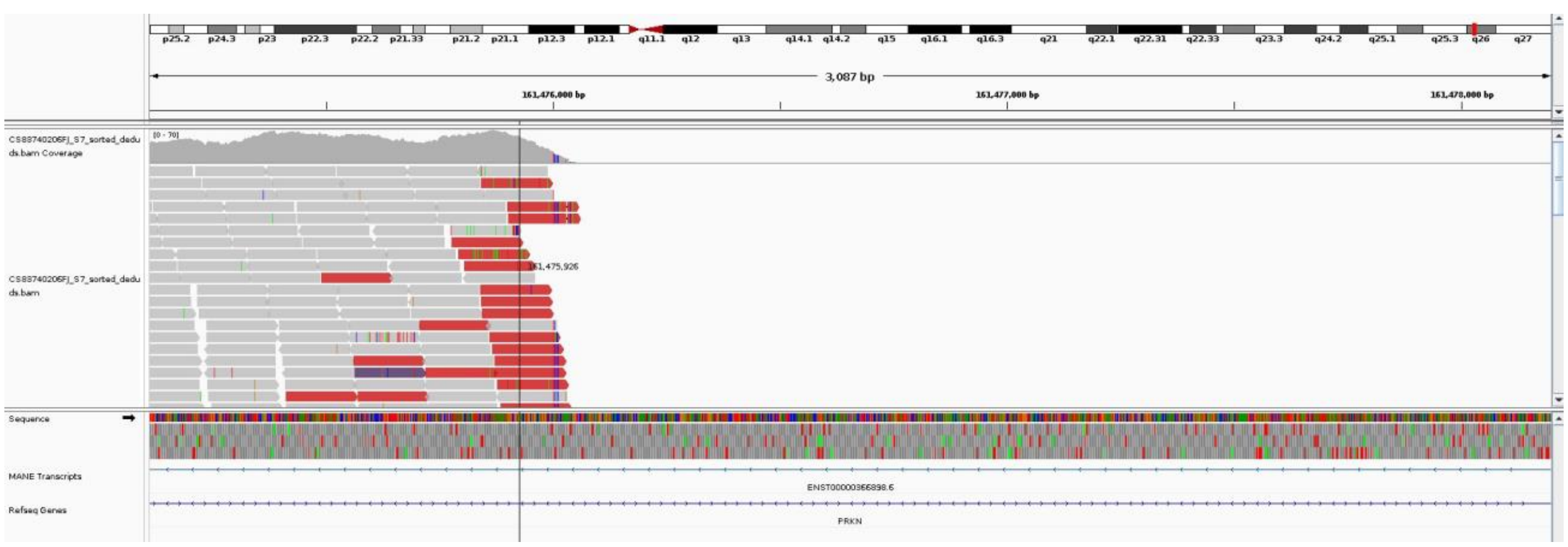

D)

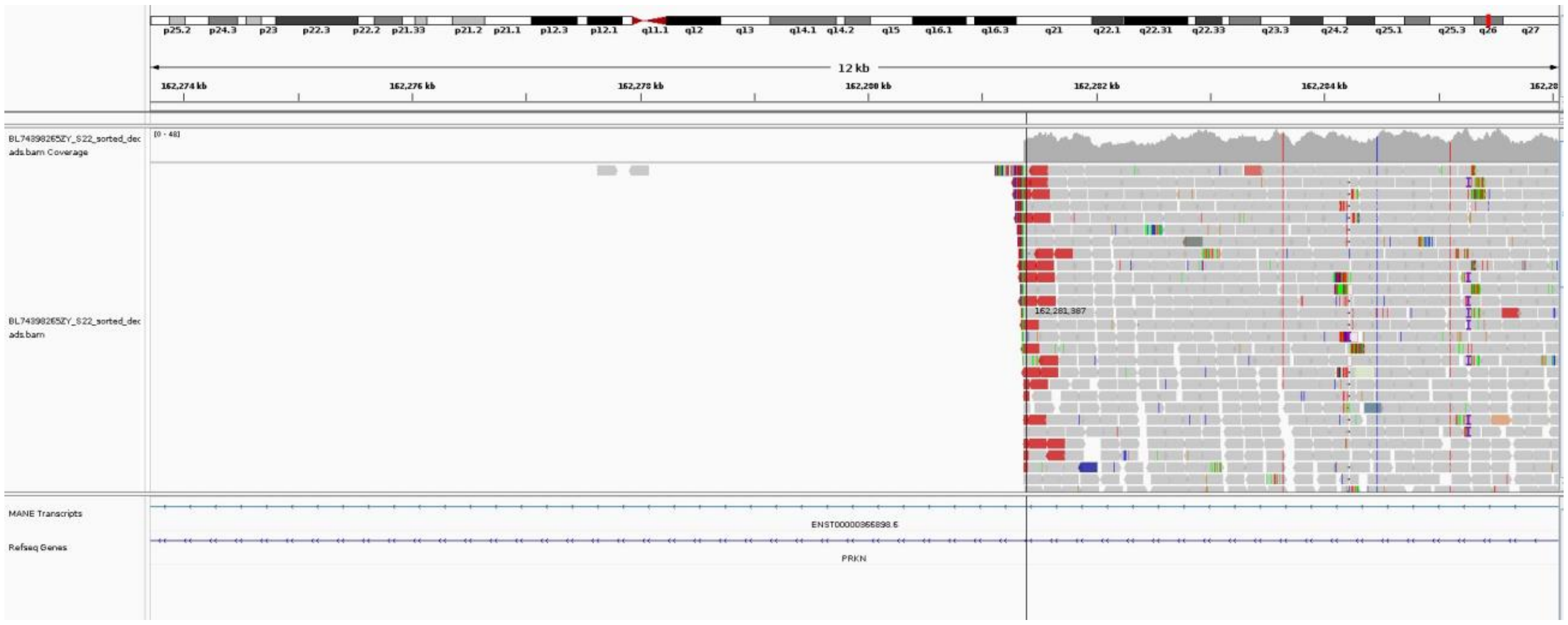
12 kb
162,274 kb
162,276 kb
162,278 kb
162,280 kb
162,282 kb
162,284 kb
BL74398265ZY_S22_sorted_dedups.bam Coverage
BL74398265ZY_S22_sorted_dedups.bam
162,281,387
MANE Transcripts
Refseq Genes
PRKN

E)

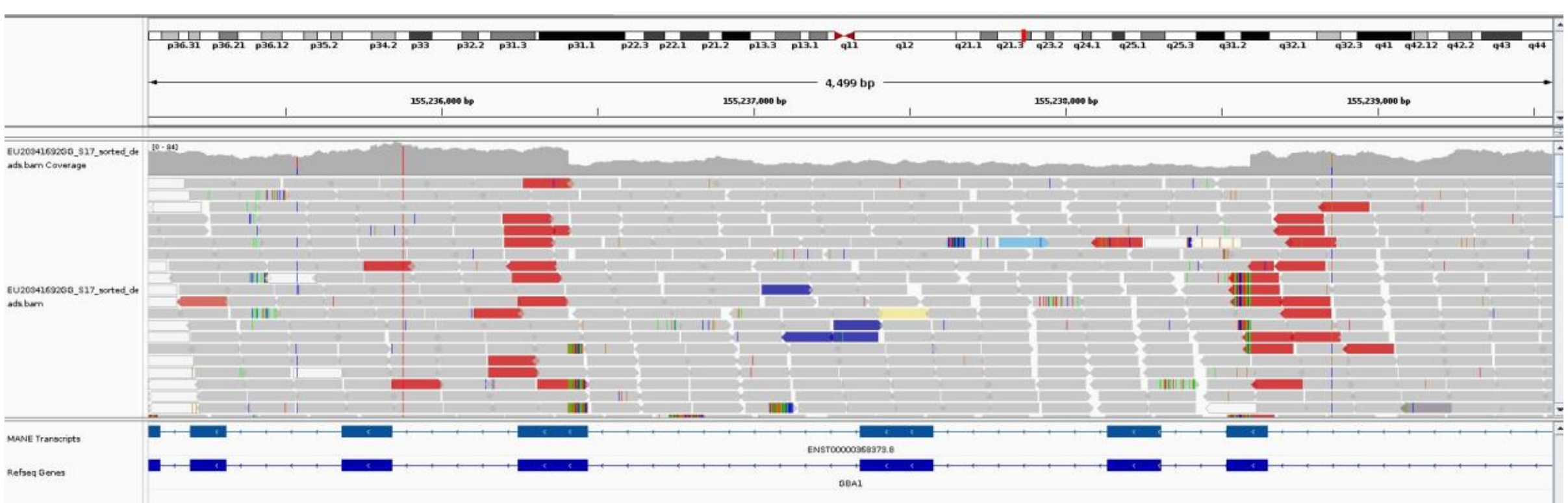
4,499 bp
155,236,000 bp
155,237,000 bp
155,238,000 bp
155,239,000 bp
MANE Transcripts
Refseq Genes

F)

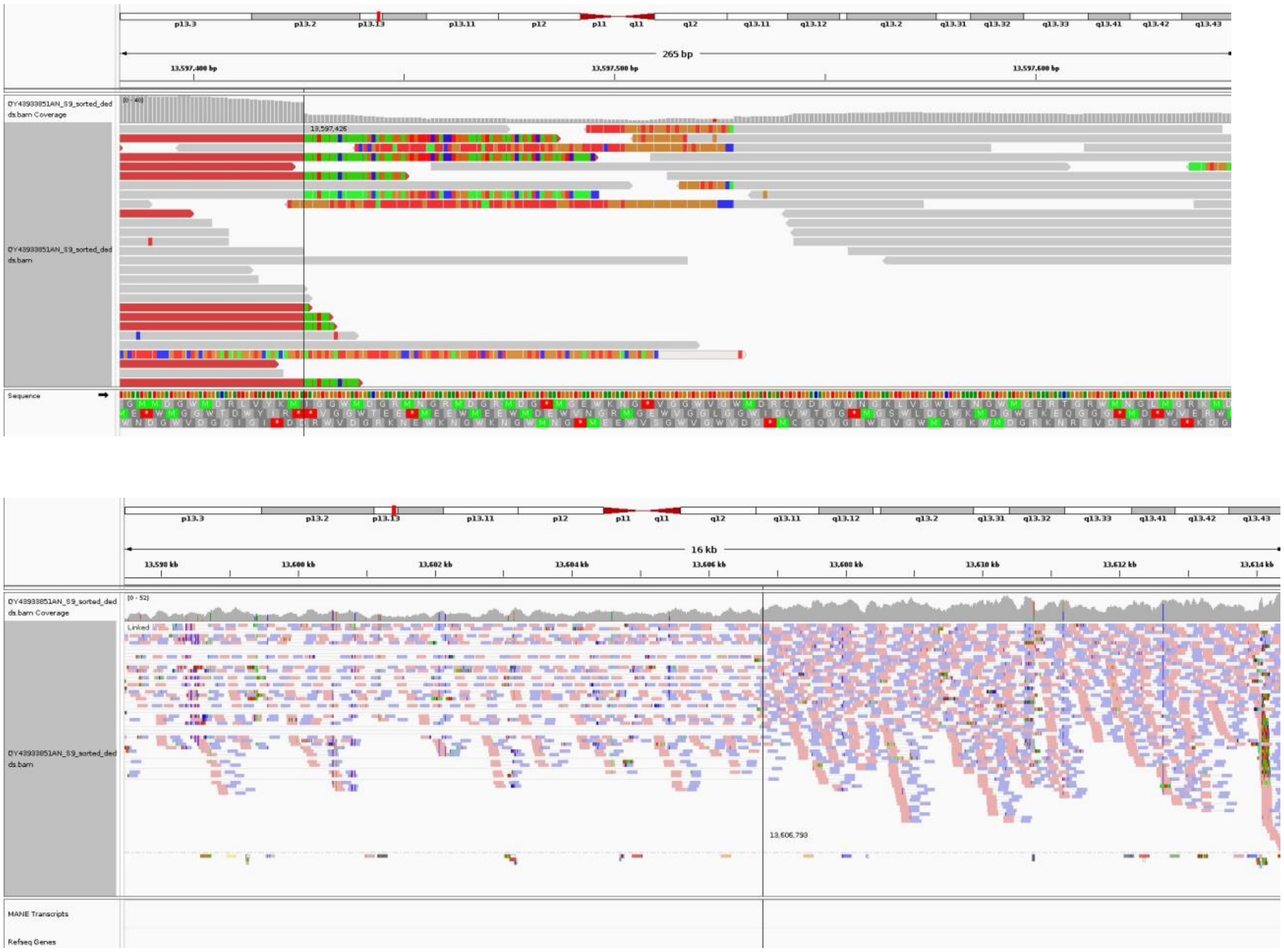

__Figure S7: IGV Snapshots of SV deletions__ Representative IGV views showing read depth and split-read evidence supporting pathogenic or candidate deletions. **(A)** Homozygous deletion spanning *PRKN* in YLOPD_83. **(B)** *PRKN* deletions in YLOPD22 and YLOPD21. **(C)** Homozygous deletion within *PRKN* in YLOPD52. **(D)** *PRKN* deletion in YLOPD108. **(E)** Heterozygous deletion encompassing *GBA1* in YLOPD31. **(F)** Heterozygous deletion within *CACNA1A* in YLOPD141.

ONT Long-Read Amplicon Sequencing: Validation of GBA1 and PLA2G6 Variants

GBA1 poses a particular challenge for short-read sequencing-based variant calling due to its high sequence homology with the nearby pseudogene *GBAP1*, located approximately 16 kb downstream on chromosome 1. The two loci share >96% sequence identity, causing short reads (150 bp) to align ambiguously between *GBA1* and *GBAP1*, and this could possibly lead to false positives. To overcome this, we performed targeted long-read amplicon sequencing using Oxford Nanopore Technologies (ONT), generating reads of ~5,700 bp that span the entire *GBA1* amplicon and distinguish the gene from its pseudogene.

For *PLA2G6*, compound heterozygosity was identified in two EOPD cases. Establishing a compound heterozygous diagnosis requires confirmation that the two pathogenic variants reside on opposite alleles (trans configuration). Since, one variant pair is separated by 3,105 bp, exceeding the insert size of the short-read WGS library, haplotype phasing from WGS-short read data alone was not possible. ONT long-read amplicon sequencing was therefore performed to determine the phase.

Amplicon Design and Primer Sequences

PCR primers were designed to amplify the complete coding region of *GBA1* (amplicon ~5,709 bp, chr1:155,213,801–155,219,510) and *PLA2G6* (amplicon ~5,325 bp, chr22:38,111,237–38,116,562), with flanking intronic sequence to ensure full coverage of all exons and splice sites. Primer sequences are provided in Table S1_Primers.

Table S1: PCR primer sequences used for ONT amplicon sequencing of GBA1 and PLA2G6.

Gene	Primer	Sequence (5’→3’)	Amplicon size	Exons
GBA1	Forward	ATGTGTCCATTCTCCATGTCTTCA	5,709 bp	Exon 3-11
GBA1	Reverse	CAGAATAAAGCCACTCACACTGAC	5,709 bp	Exon 3-11
PLA2G6	Forward	CAAGATGAAGTTGAAGATTGTGGC	5,325 bp	Exon 13-17
PLA2G6	Reverse	CCTTTTTCTAACGTCTGCAGTGTG	5,325 bp	Exon 13-17

Table S2: PCR primer sequences used for Sanger sequencing of GBA1

Gene	Primer	Sequence (5’→3’)	Amplicon size	Exons
GBA1	GBA1-FP1	TCCTAAAGTTGTCACCCATACATG	1243 bp	Exon 2
GBA1	GBA1-RP1	ACTGAACACGGTTTCAAAATTCC	1243 bp	Exon 2

Library Preparation and Sequencing

PCR amplicons were verified by agarose gel electrophoresis. Sequencing was done on the Oxford Nanopore Technologies (ONT) MinION platform. Reads were basecalled using Dorado. ONT ligation sequencing adapters were removed during basecalling by Dorado. Read quality was assessed using NanoStat.

Reads shorter than 90% of the expected amplicon length were removed using seqkit, retaining reads ≥5,138 bp for *GBA1* (90% of 5,709 bp), ≥6,288 bp for YLOPD20 (90% of 6,987 bp), and

≥4,792 bp for *PLA2G6* (90% of 5,325 bp) to exclude adapter dimers, chimeric fragments, and degraded DNA. Filtered reads were aligned to the GRCh38 reference genome (GCA_000001405.15, no-alt contigs) using minimap2 v2.27 with the ONT long-read preset (*-ax map-ont*) and the *--MD* flag. Read group tags were assigned per sample. Alignments were sorted and indexed using samtools v1.21.

Variants were called using GATK HaplotypeCaller v4.6.1 in GVCF mode with *--pcr-indel-model NONE* and *--max-reads-per-alignment-start 0*. Variants were hard-filtered for SNPs (QD < 2.0, FS > 60.0, MQ < 40.0) and indels (QD < 2.0, FS > 200.0). For sample YLOPD31, which carries a known heterozygous structural variant in GBA1, structural variant calling was additionally performed using Sniffles2 v2.7.3 on unfiltered reads with --minsupport 3. For the two *PLA2G6* cases, haplotype phasing was performed using WhatsHap v2.3 (*--indels --reference*). Reads were haplotagged using WhatsHap haplotag and visualised in IGV v2.16.2 with reads coloured by HP haplotype tag.

***GBA1* Variant Validation**

All 13 *GBA1* SNVs and the heterozygous structural variant (sample YLOPD31) were confirmed by ONT amplicon sequencing (Figure S8). Mapping quality was MAPQ = 60 for 99.9-100% of primary reads in 13 of 14 samples. Sample YLOPD31, which carries a heterozygous structural variant, showed 89.1% MAPQ60 reads; the reduced proportion is attributable to split-read alignments at the deletion breakpoints rather than pseudogene misalignment. No multimapping alignments (MAPQ = 0) were detected in any sample, confirming unambiguous alignment to the *GBA1* rather than *GBAP1* pseudogene.

ONT amplicon sequencing confirmed *GBA1* variants in 13 of 14 samples with sufficient sequencing depth. All confirmed variants showed allele balance consistent with heterozygosity (VAF range 44.8–50.8%), with the exception of YLOPD208 which showed a homozygous ALT allele at chr1:155,238,596 C>T (3,018 ALT reads, 0 reference reads, VAF = 100%), concordant with the homozygous genotype called by WGS.

Critically, ONT sequencing corrected the ALT allele assignment at position chr1:155,235,252 in three of four samples carrying a variant at this locus. WGS called A>G in YLOPD_62 (confirmed by ONT, AD: 867/793) and YLOPD128, and A>C in YLOPD29 and YLOPD184. However, ONT long-read sequencing revealed: YLOPD128 carries A>C (AD: 1,511/1,257), not A>G as called by WGS; YLOPD29 carries A>G (AD: 883/650), not A>C; and YLOPD184 carries A>G (AD: 2,436/1,910), not A>C. All four ONT calls were high-confidence (FILTER=PASS, GQ=99, DP=1,536–4,353). These discrepancies are consistent with well-documented short-read misalignment between *GBA1* and the *GBAP1* pseudogene, which shares >96% sequence identity. Short reads (150 bp) lack sufficient flanking sequence context to unambiguously distinguish the two loci, leading to incorrect ALT allele assignment. ONT reads spanning the full 5,709 bp amplicon resolve this ambiguity definitively, demonstrating that long-read sequencing is essential for accurate variant characterisation in *GBA1*.

For sample YLOPD31, Sniffles2 v2.7.3 was run on unfiltered reads (prior to the 90% length filter) to capture reads from both haplotypes, including the shorter reads (~3,500 bp) arising from the deleted allele. Sniffles2 identified a 2,185 bp heterozygous deletion at chr1:155,236,405–155,238,590 (SVTYPE=DEL; FILTER=PASS; PRECISE). Of 11,956 primary mapped reads, 6,071 (50.8%) supported the deletion allele, consistent with a heterozygous variant. The

remaining 5,885 reads (49.2%) represented the intact reference haplotype. Representative IGV screenshots showing ONT read pileups at each variant position are provided in Figure S8.

PLA2G6 Compound Heterozygosity

In both YLOPD238 and YLOPD50, WhatsHap read-backed phasing confirmed that the two *PLA2G6* pathogenic variants are in trans configuration (on opposite haplotypes), establishing compound heterozygosity. Both variant pairs were resolved within a single phase block spanning the full amplicon length. IGV snapshots of the phased haplotypes are provided in Figure S10. The phased genotypes are summarised below.

Table S3. Phased genotypes for PLA2G6 compound heterozygous cases. GT: phased genotype (| notation); HP: haplotype assignment; Trans: variants on opposite haplotypes confirming compound heterozygosity.

Case	Variant 1	GT	HP	Variant 2	GT	HP	Phase
YLOPD238	c.2341G>A	1|0	HP1	c.2239C>T	0|1	HP2	Trans
YLOPD50	c.2222G>A	0|1	HP2	c.1898C>T	1|0	HP1	Trans

IGV snapshots for GBA1 validation – using long read ONT

A)

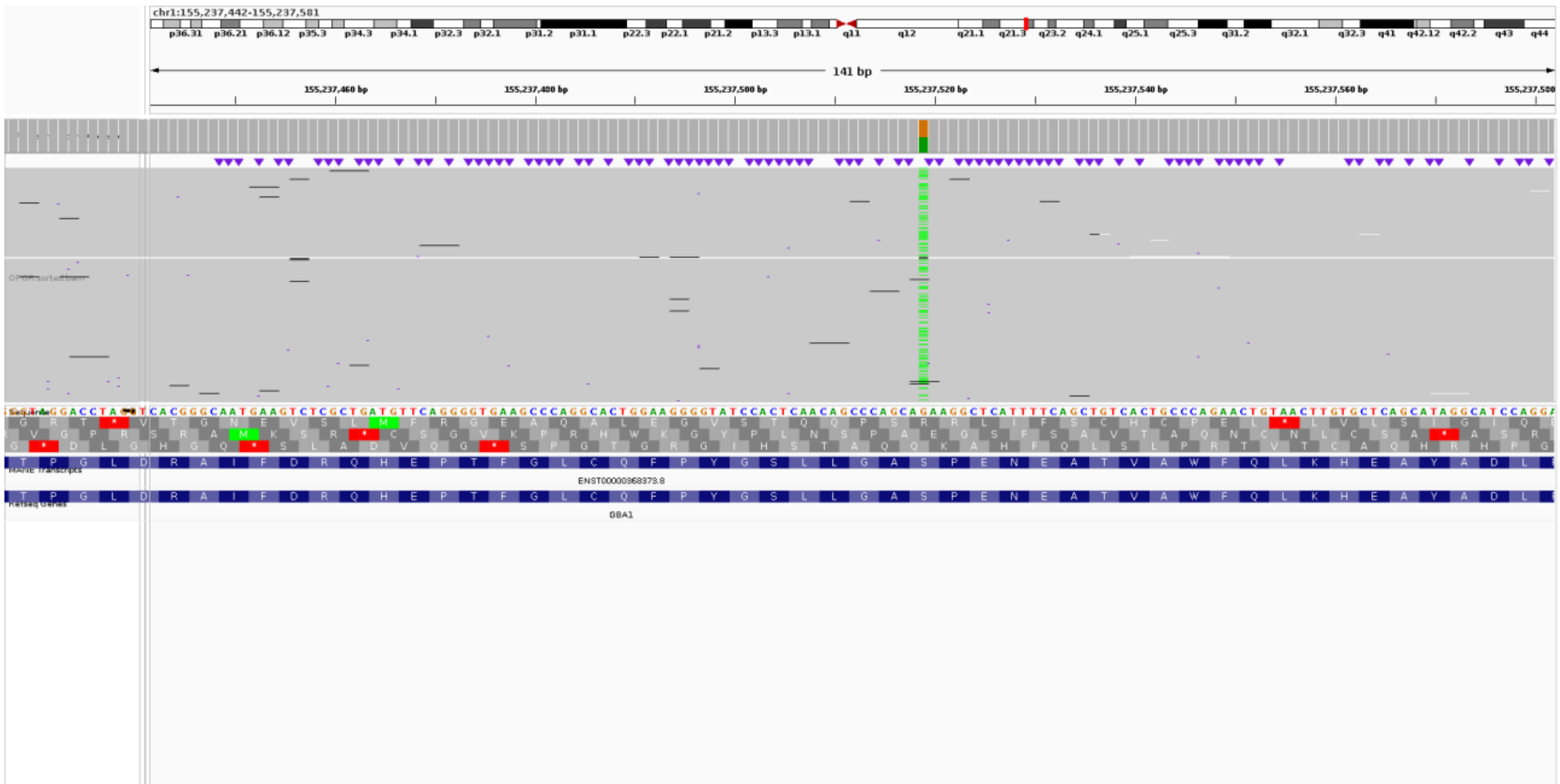

B)

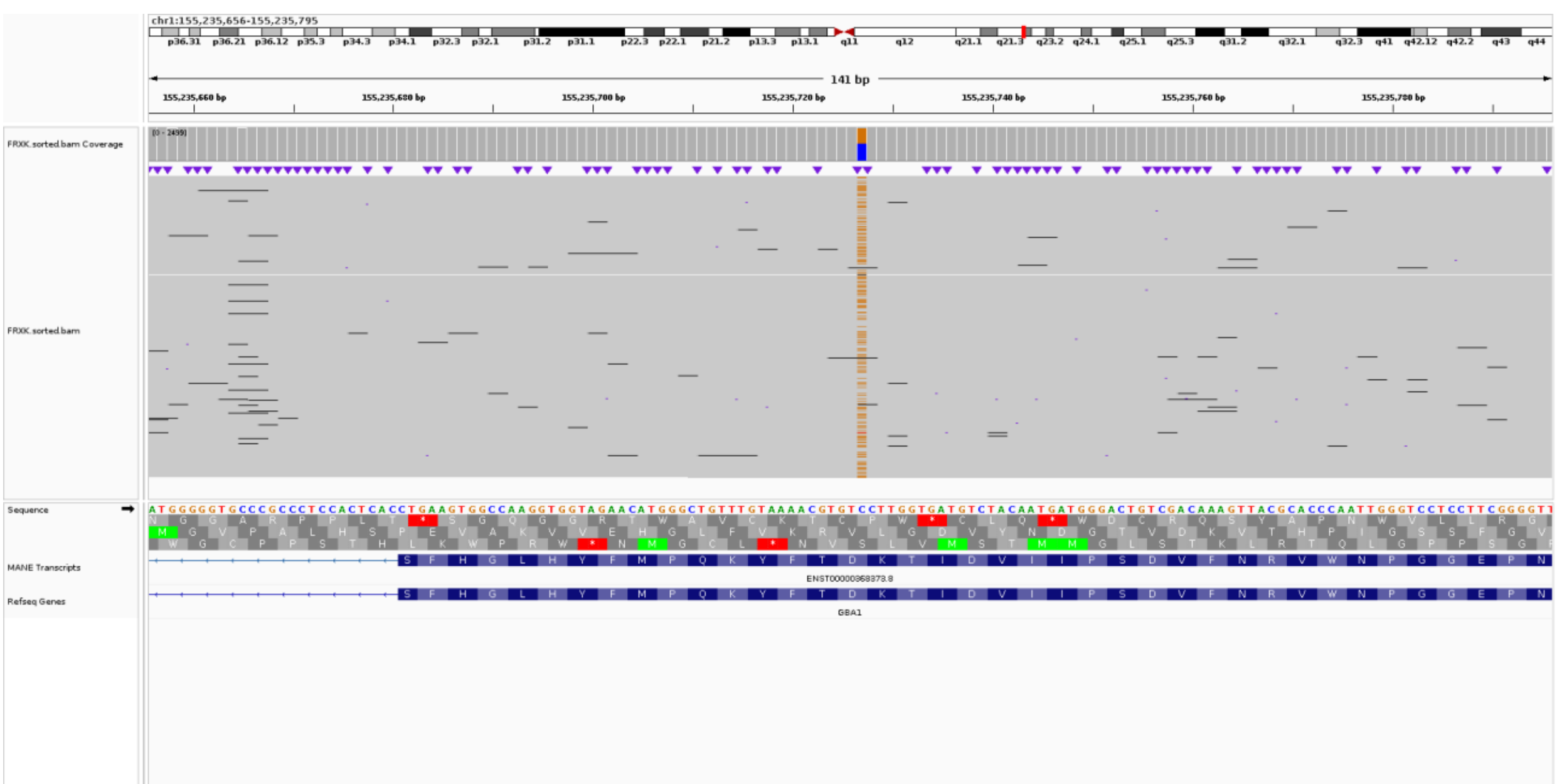

C)

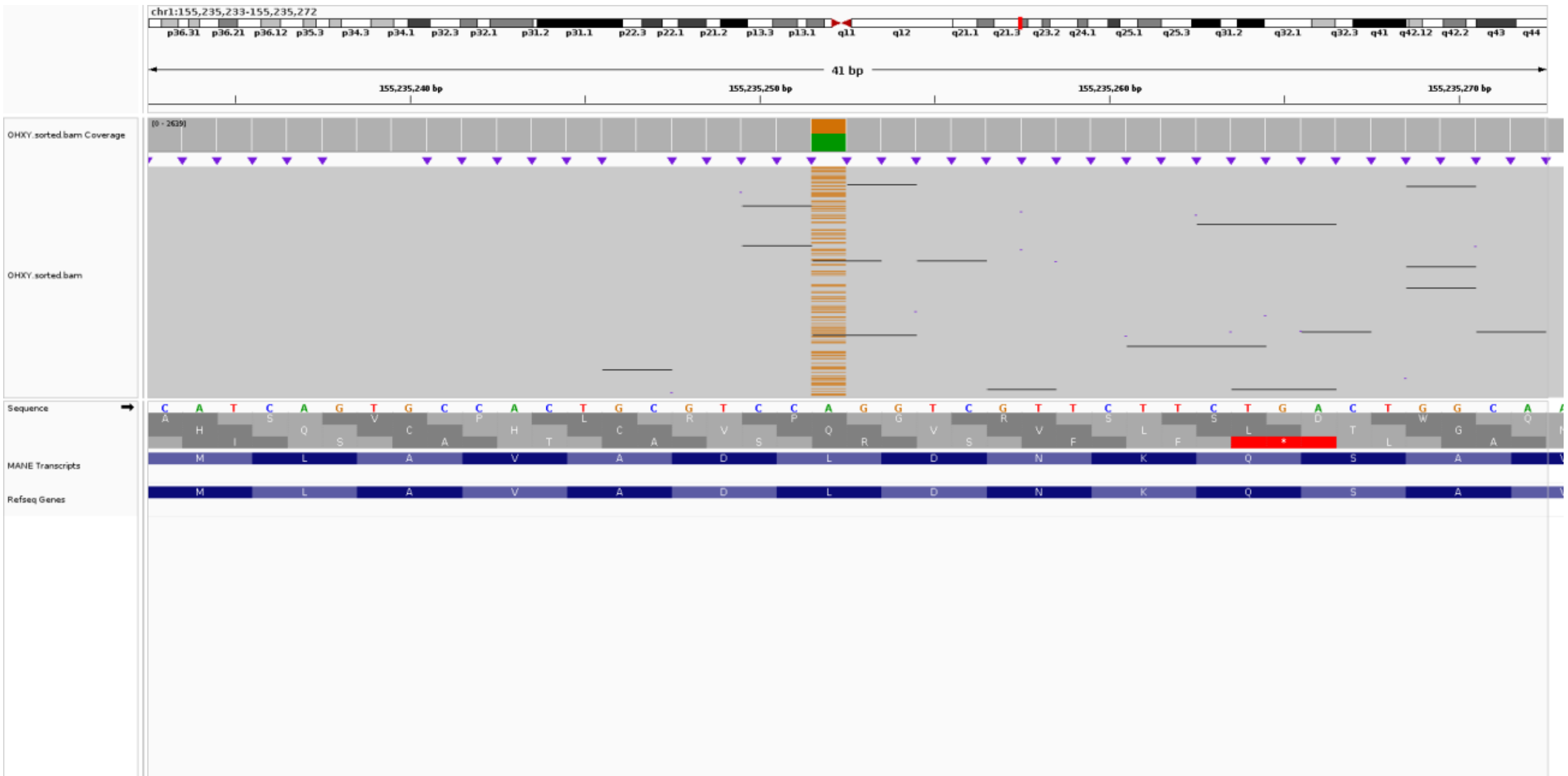

chr1:155,235,233-155,235,272
41 bp
155,235,240 bp
155,235,250 bp
155,235,260 bp
155,235,270 bp
OHXY.sorted.bam Coverage
OHXY.sorted.bam
Sequence
MANE Transcripts
Refseq Genes

D)

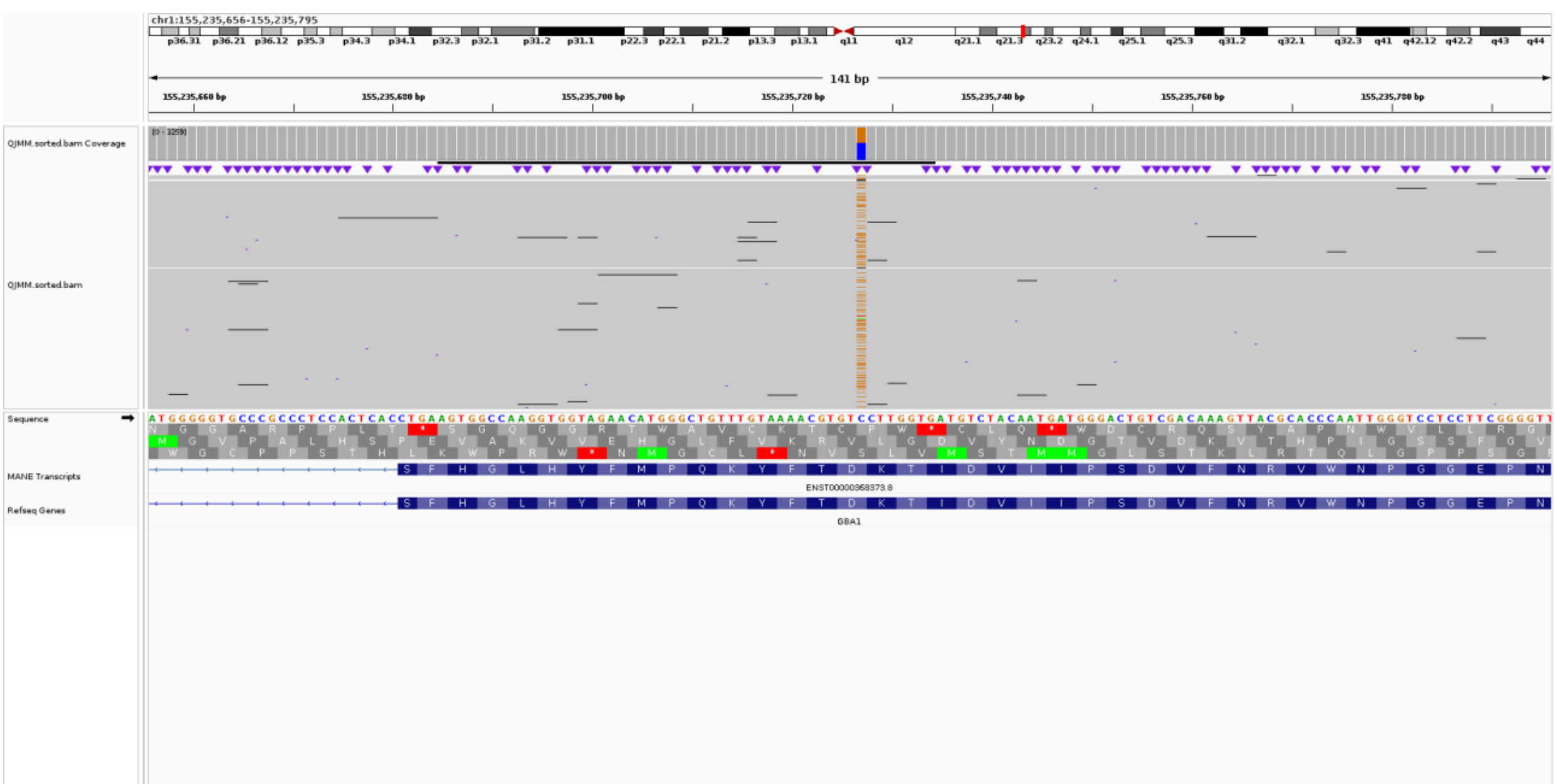

chr1:155,235,656-155,235,795
141 bp
QJMM.sorted.bam Coverage
QJMM.sorted.bam
Sequence
MANE Transcripts
Refseq Genes
GBA1

E)

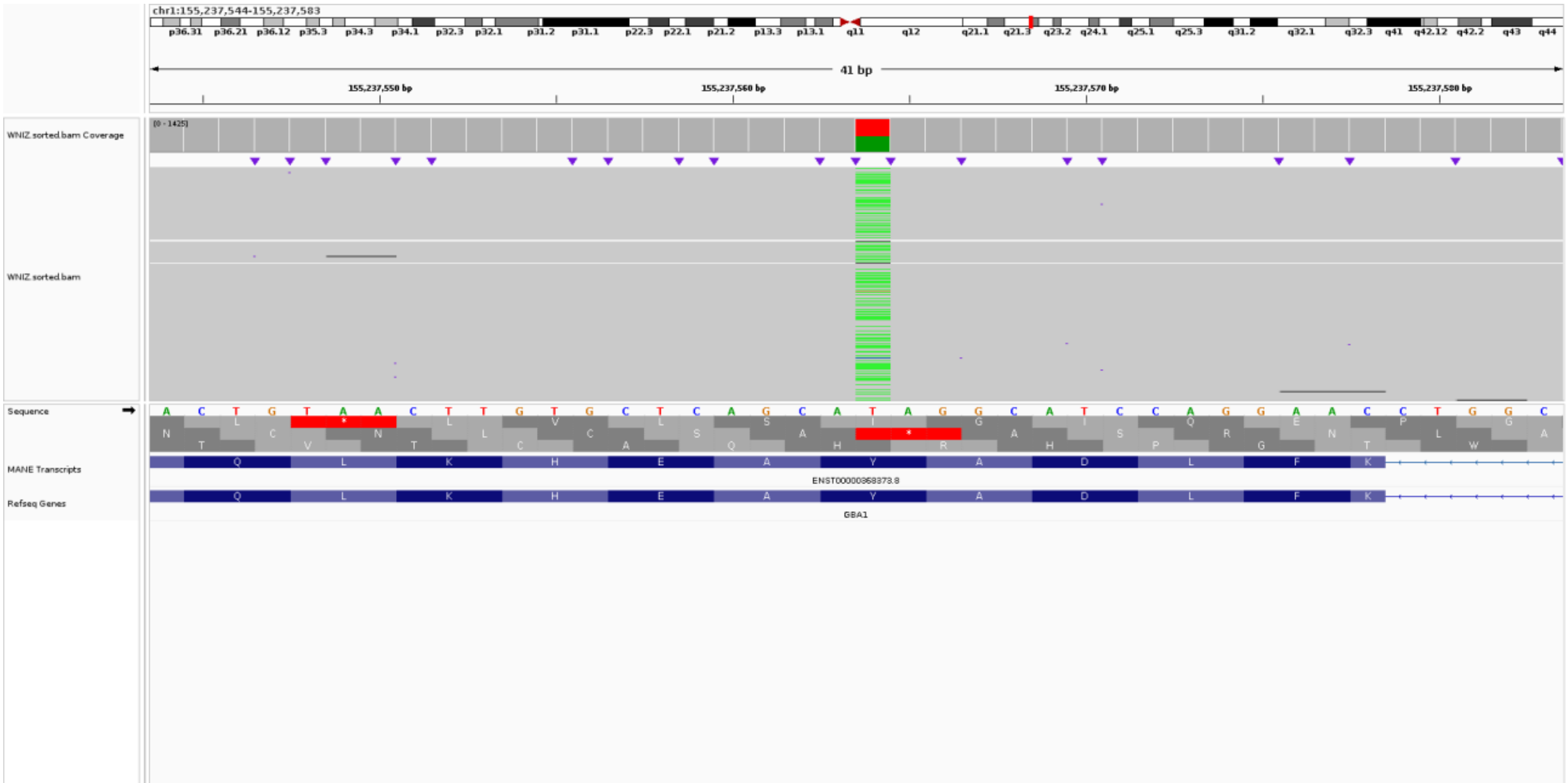
chr1:155,237,544-155,237,583
41 bp
155,237,550 bp
155,237,560 bp
155,237,570 bp
155,237,580 bp
WNIZ.sorted.bam Coverage
WNIZ.sorted.bam
Sequence
MANE Transcripts
Refseq Genes
ENST00000368373.8
GBA1

F)

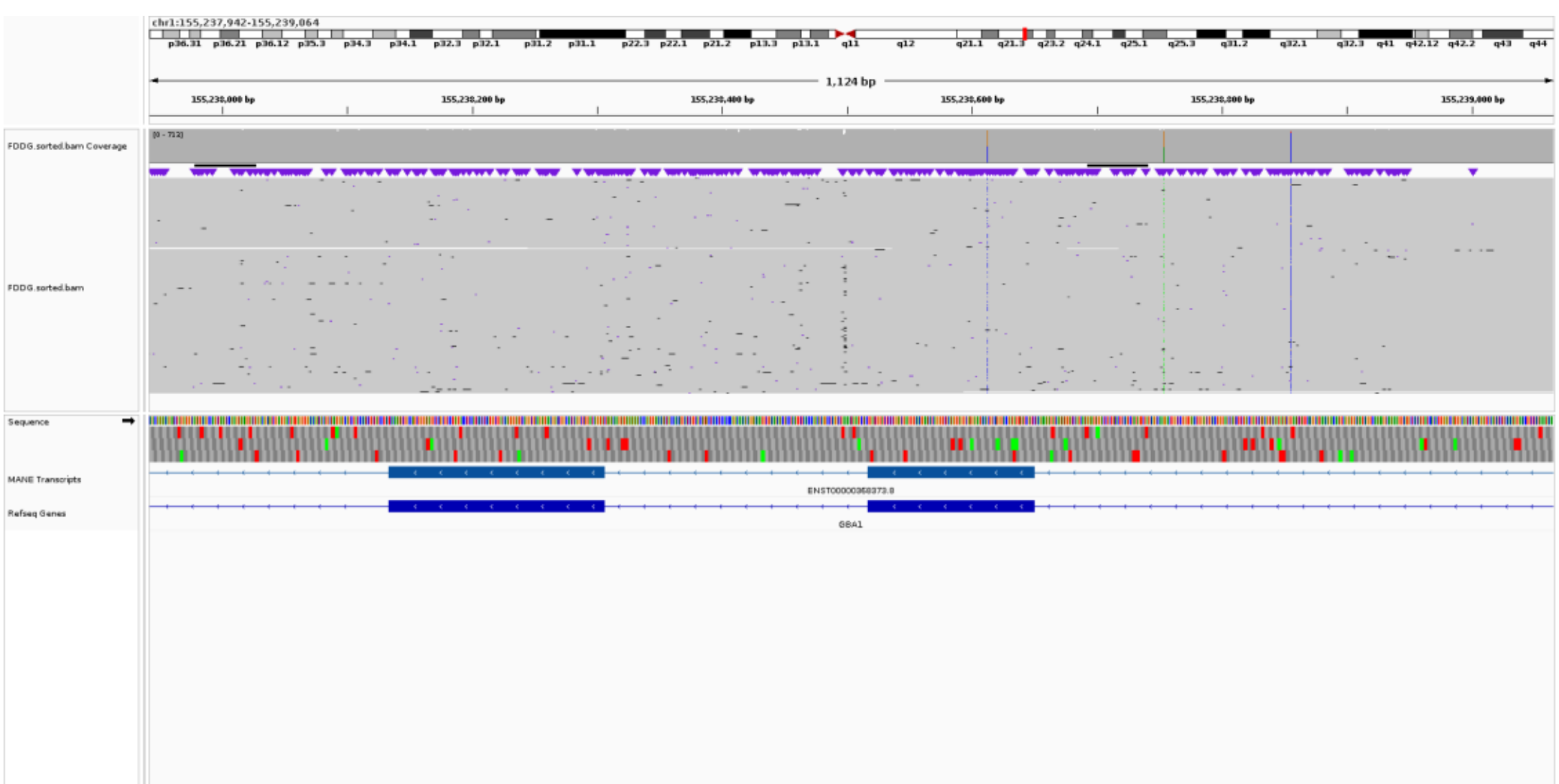
chr1:155,237,942-155,239,064
1,124 bp
FDDG.sorted.bam Coverage
FDDG.sorted.bam
Sequence
MANE Transcripts
Refseq Genes
ENST00000368373.8
GBA1

G)

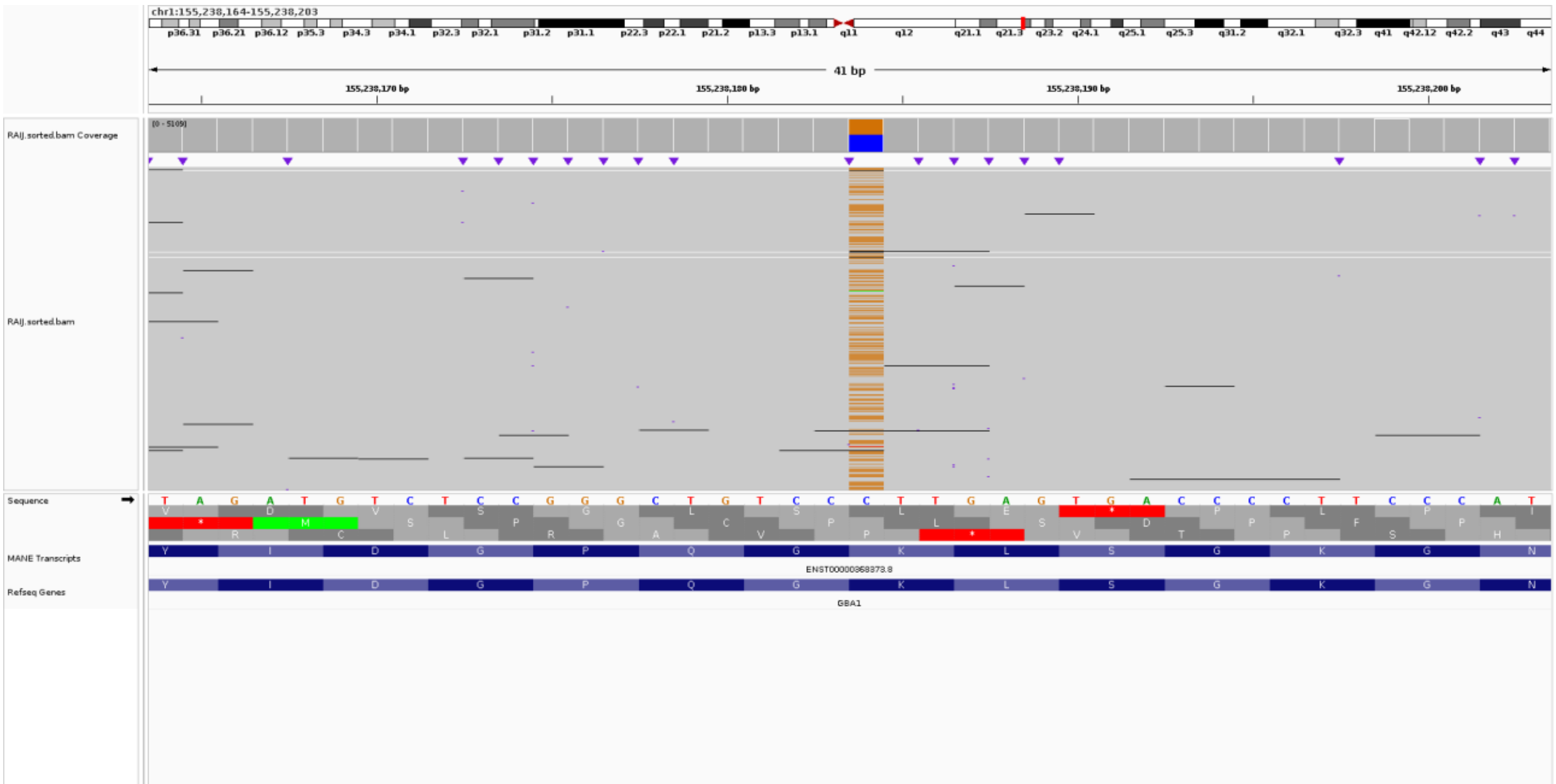
chr1:155,238,164-155,238,203
41 bp
155,238,170 bp
155,238,180 bp
155,238,190 bp
155,238,200 bp
RAJJ.sorted.bam Coverage
RAJJ.sorted.bam
Sequence
MANE Transcripts
ENST00000368373.8
Refseq Genes
GBA1

H)

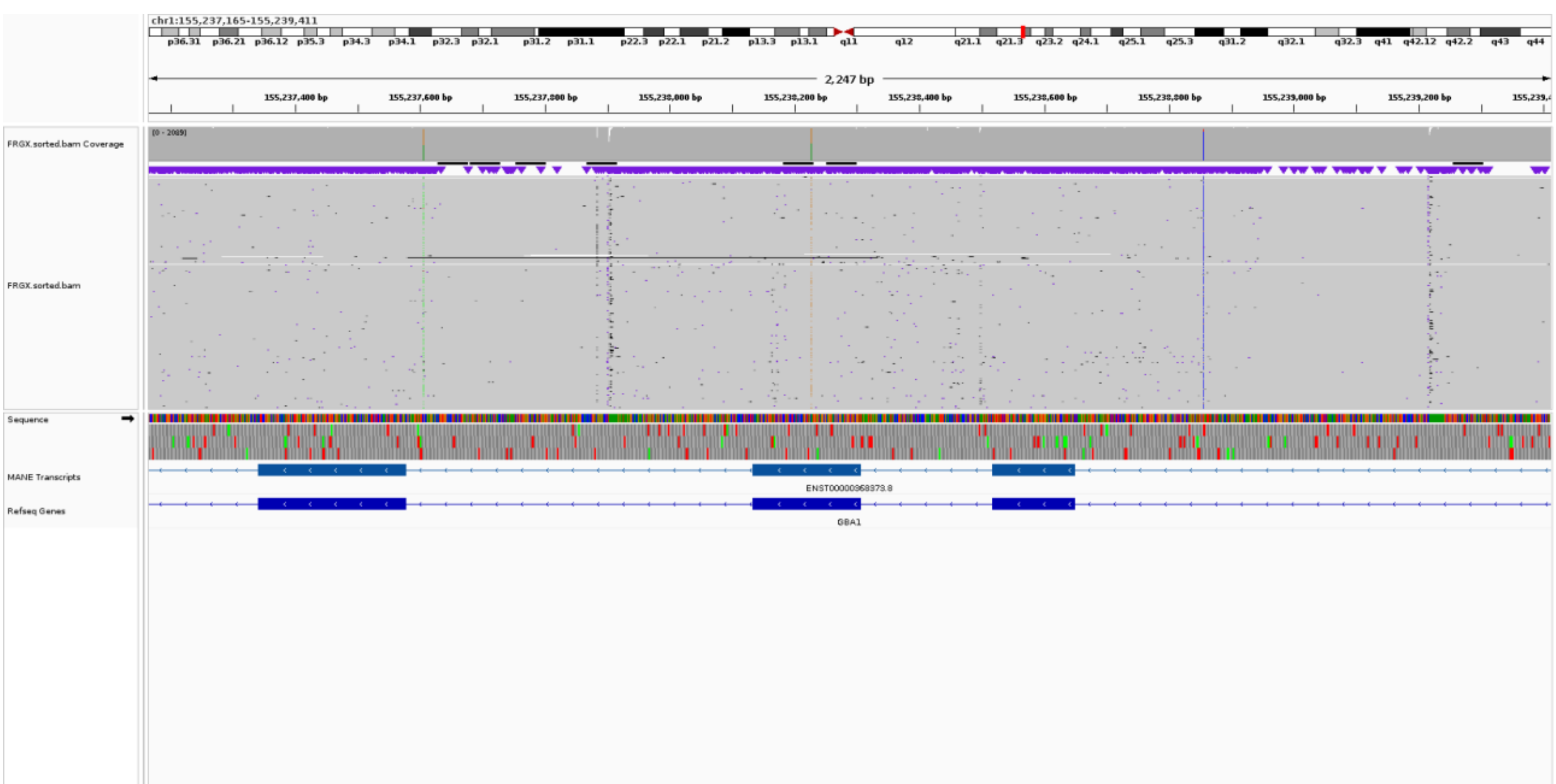
chr1:155,237,165-155,239,411
2,247 bp
FRGX.sorted.bam Coverage
FRGX.sorted.bam
Sequence
MANE Transcripts
ENST00000368373.8
Refseq Genes
GBA1

I)

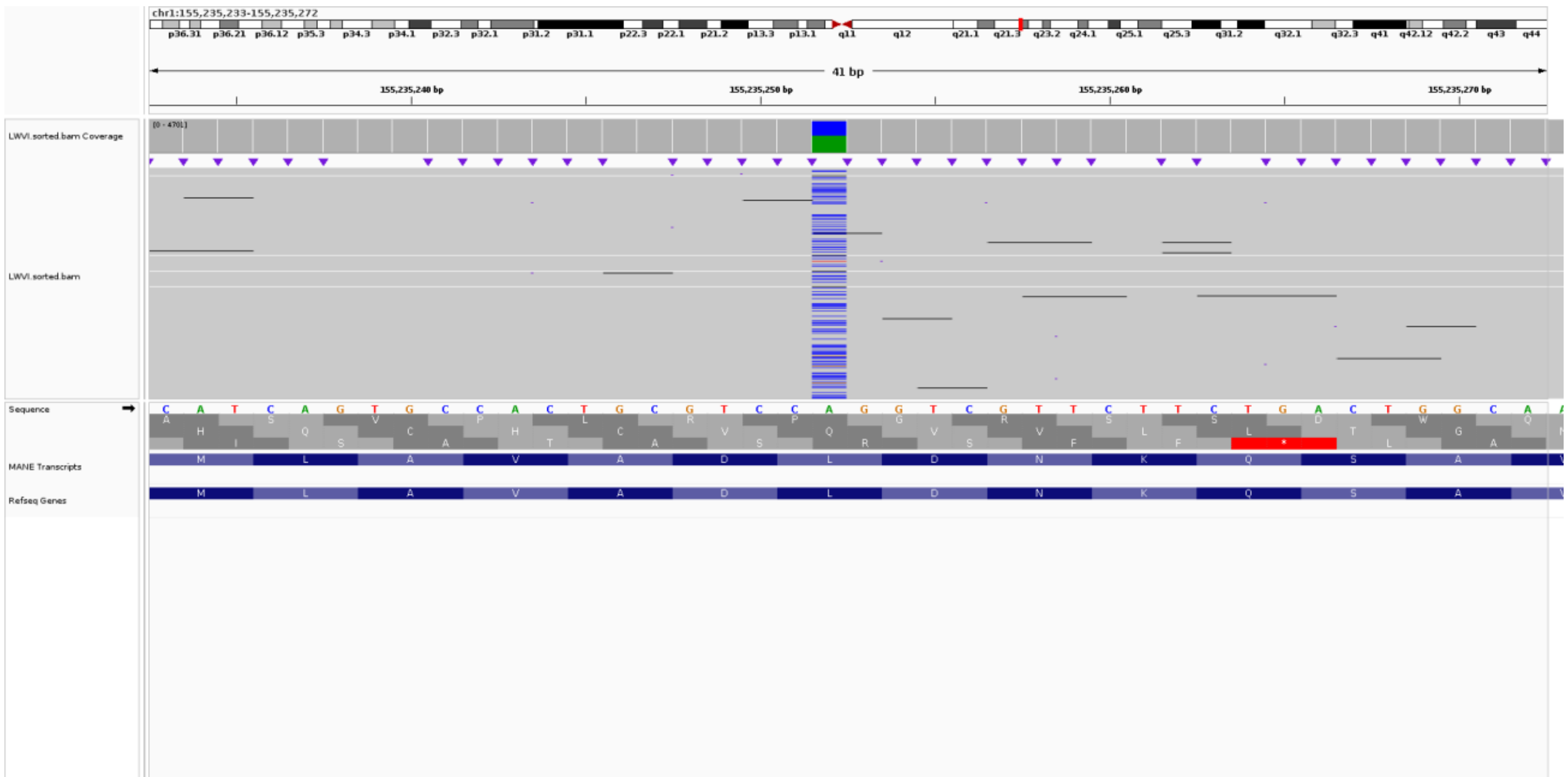
chr1:155,235,233-155,235,272
41 bp
155,235,240 bp
155,235,250 bp
155,235,260 bp
155,235,270 bp
LWVI.sorted.bam Coverage
LWVI.sorted.bam
Sequence
MANE Transcripts
Refseq Genes

J)

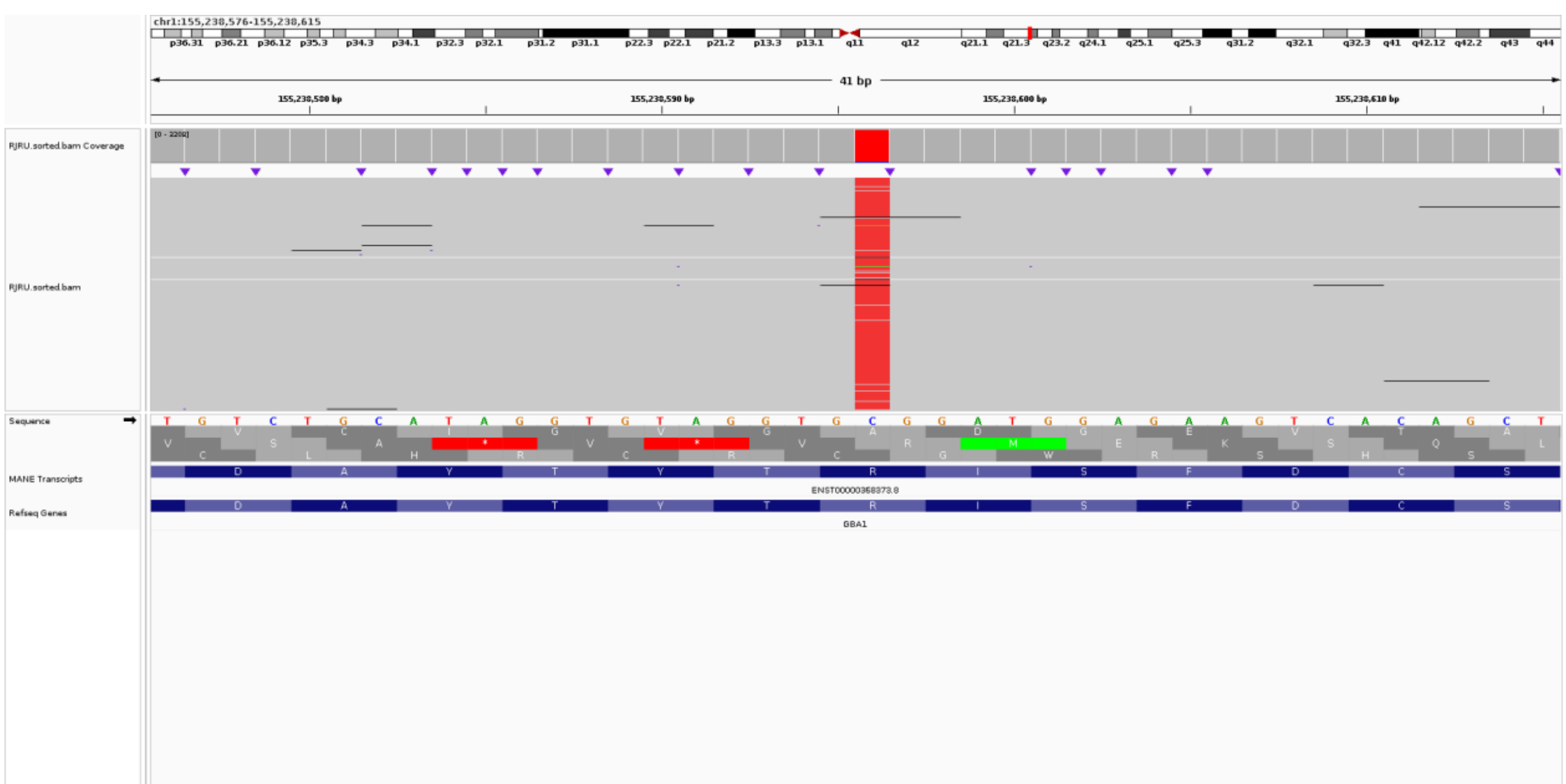
chr1:155,238,576-155,238,615
41 bp
155,238,580 bp
155,238,590 bp
155,238,600 bp
155,238,610 bp
PJRU.sorted.bam Coverage
PJRU.sorted.bam
Sequence
MANE Transcripts
Refseq Genes
GBA1

K)

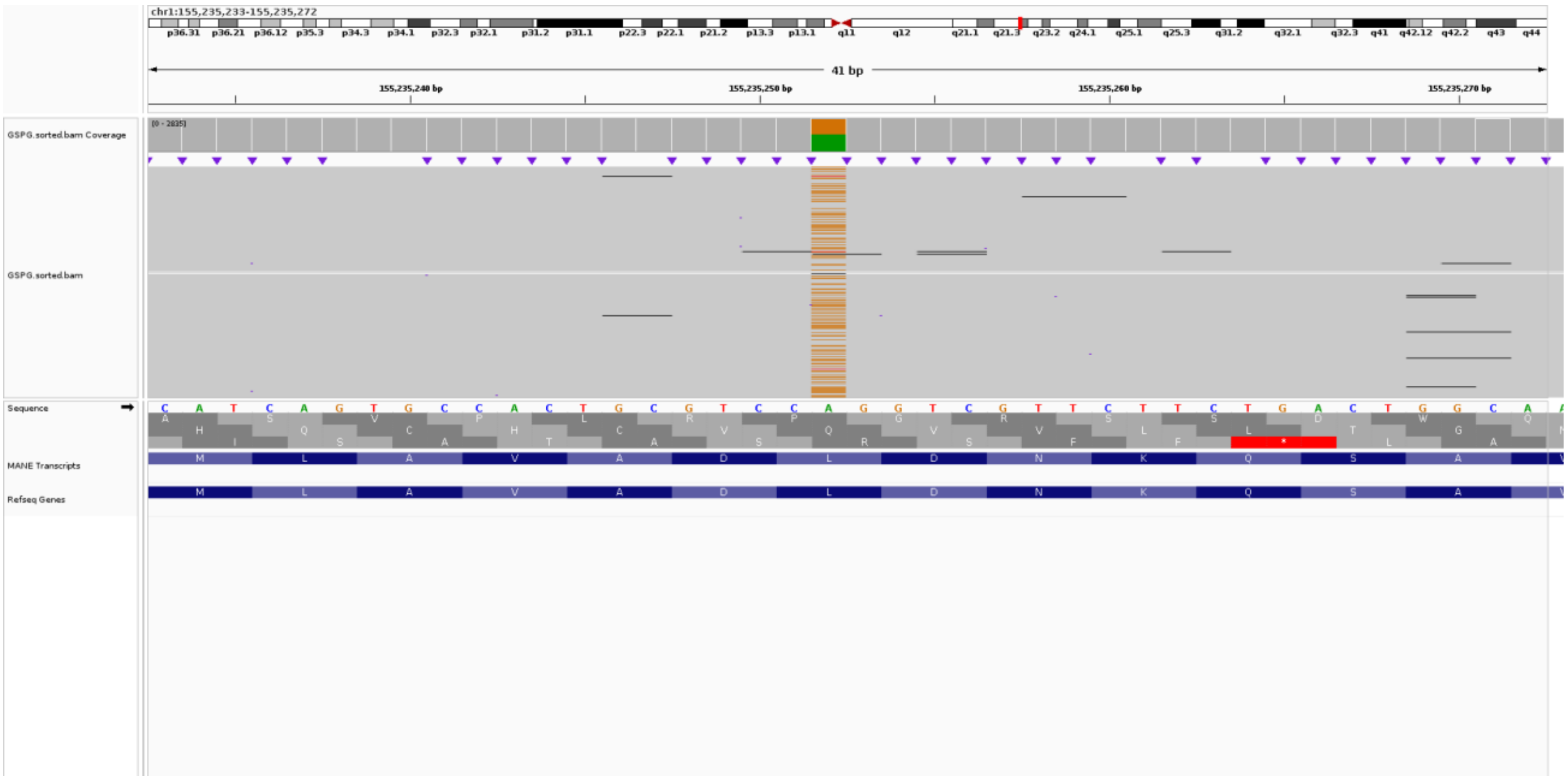
chr1:155,235,233-155,235,272
41 bp
155,235,240 bp
155,235,250 bp
155,235,260 bp
155,235,270 bp
GSPG.sorted.bam Coverage
GSPG.sorted.bam
Sequence
MANE Transcripts
Refseq Genes

L)

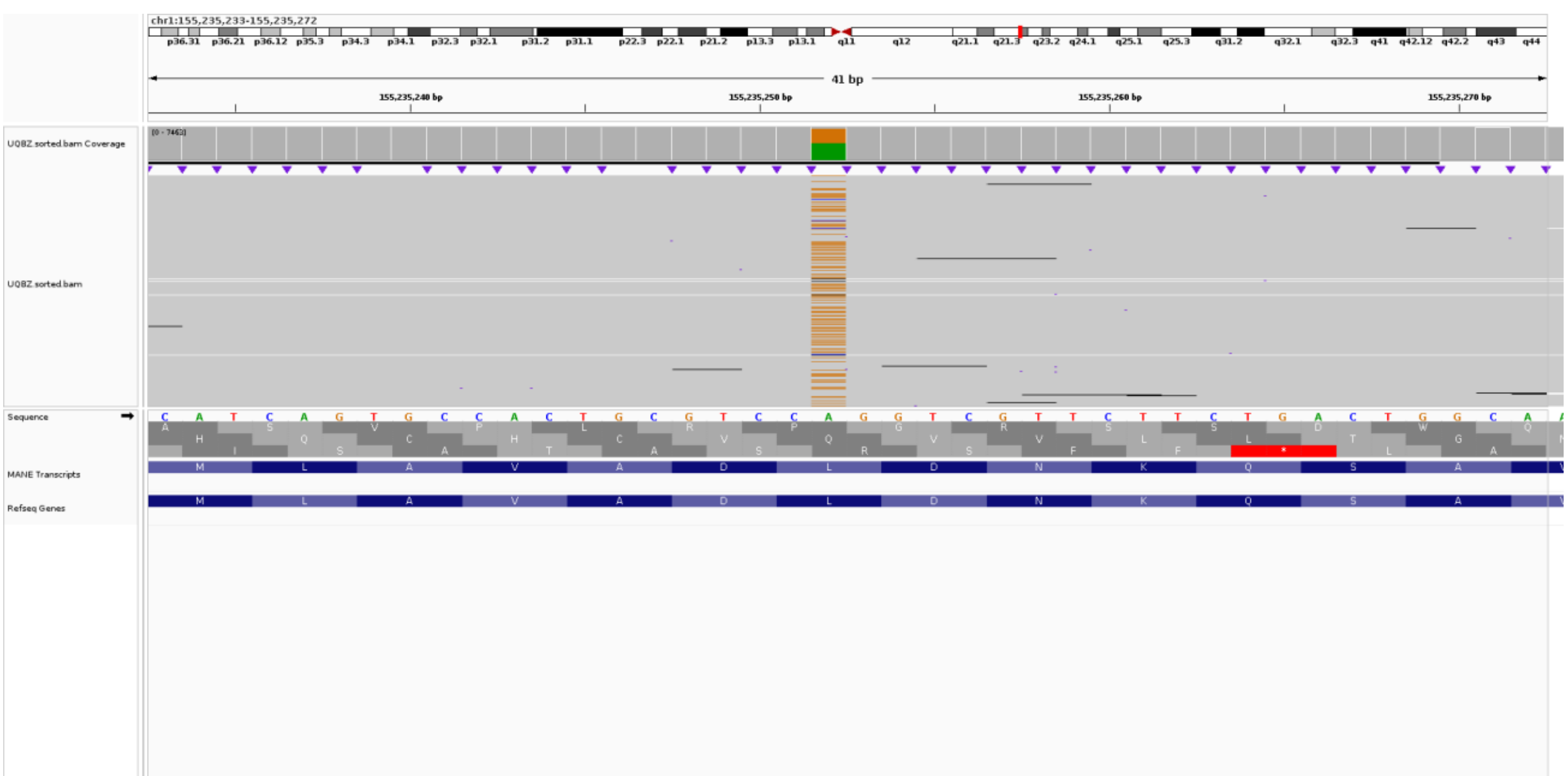
chr1:155,235,233-155,235,272
41 bp
155,235,240 bp
155,235,250 bp
155,235,260 bp
155,235,270 bp
UQBZ.sorted.bam Coverage
UQBZ.sorted.bam
Sequence
MANE Transcripts
Refseq Genes

M)

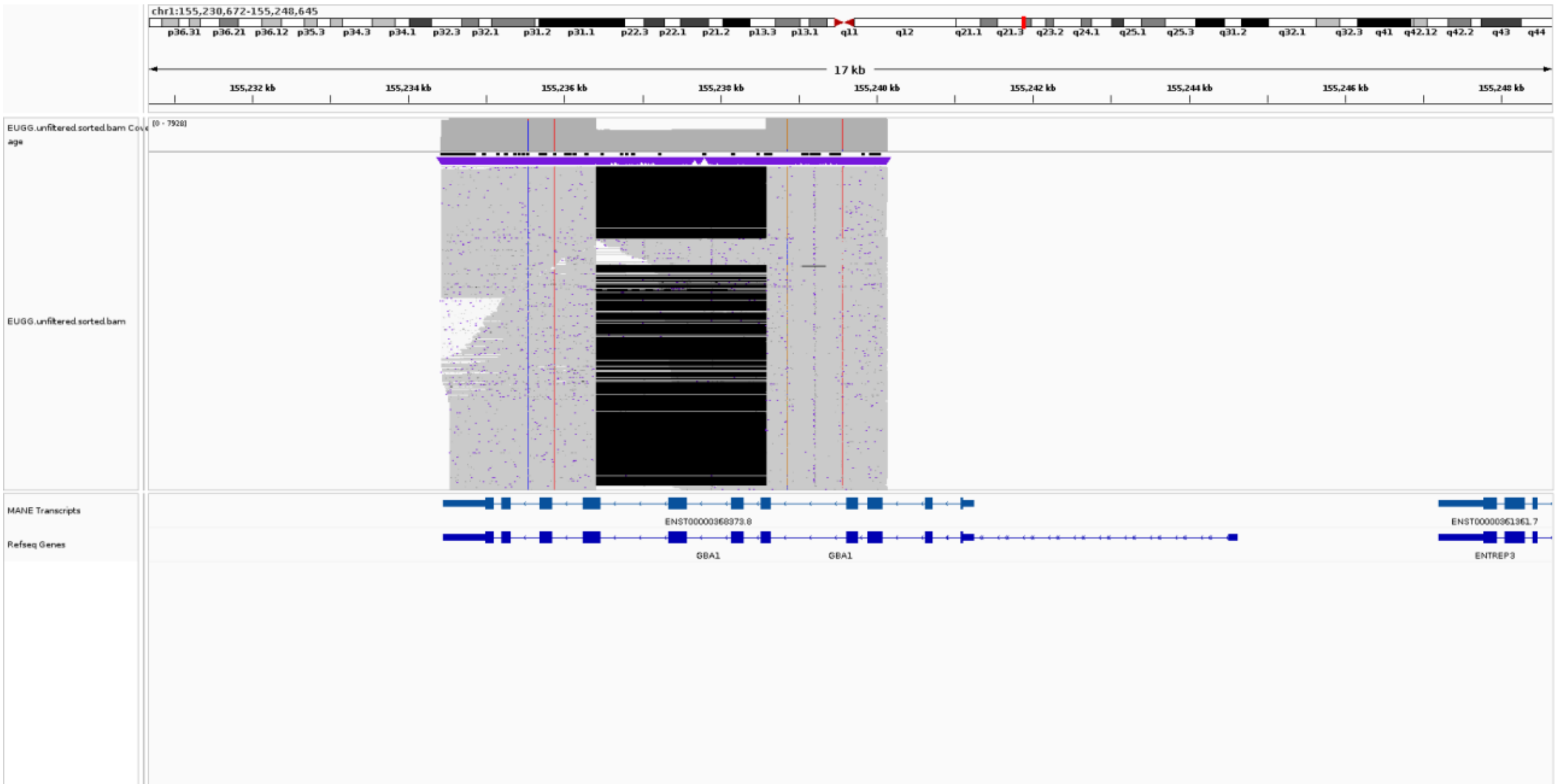

Figure S8. ONT amplicon sequencing validates GBA1 variants in YLOPD participants. IGV snapshots of Oxford Nanopore Technology (ONT) long-read amplicon sequencing confirming *GBA1* variants. **(A)** YLOPD227, **(B)**YLOPD152, **(C)** YLOPD29, **(D)** YLOPD193, **(E)** YLOPD02, **(F)** YLOPD40, **(G)** YLOPD96, **(H)** YLOPD209, **(I)**YLOPD128, **(J)** YLOPD208, **(K)** YLOPD62, **(L)** YLOPD184, **(M)** YLOPD31 (heterozygous SV deletion). Read pileups are shown relative to GRCh38/hg38.

YLOPD20: ONT sequencing failure and sanger sequencing confirmation

For sample YLOPD20 ONT amplicon sequencing failed due to DNA degradation prior to library preparation. The sample yielded 126,028 total reads, but the median read length was only 214 bp (expected ~6,987 bp for the YLOPD20-specific amplicon), with a N50 of 393 bp (5.6% of the expected amplicon size). Only 8 reads passed the 90% length filter. The high sequencing quality

score (median Q24.7) confirmed that the sequencer and flow cell were functioning correctly; the failure was attributable to shearing of the input DNA prior to or during library preparation. The sample has been flagged for resequencing. The *GBA1* variant identified by WGS in YLOPD20 was independently confirmed by Sanger sequencing. Primers used for amplifying the region is in Table_S2_Primers. Sanger chromatograms showed a clear heterozygous peak at the variant position, consistent with the WGS genotype call (Figure S9).

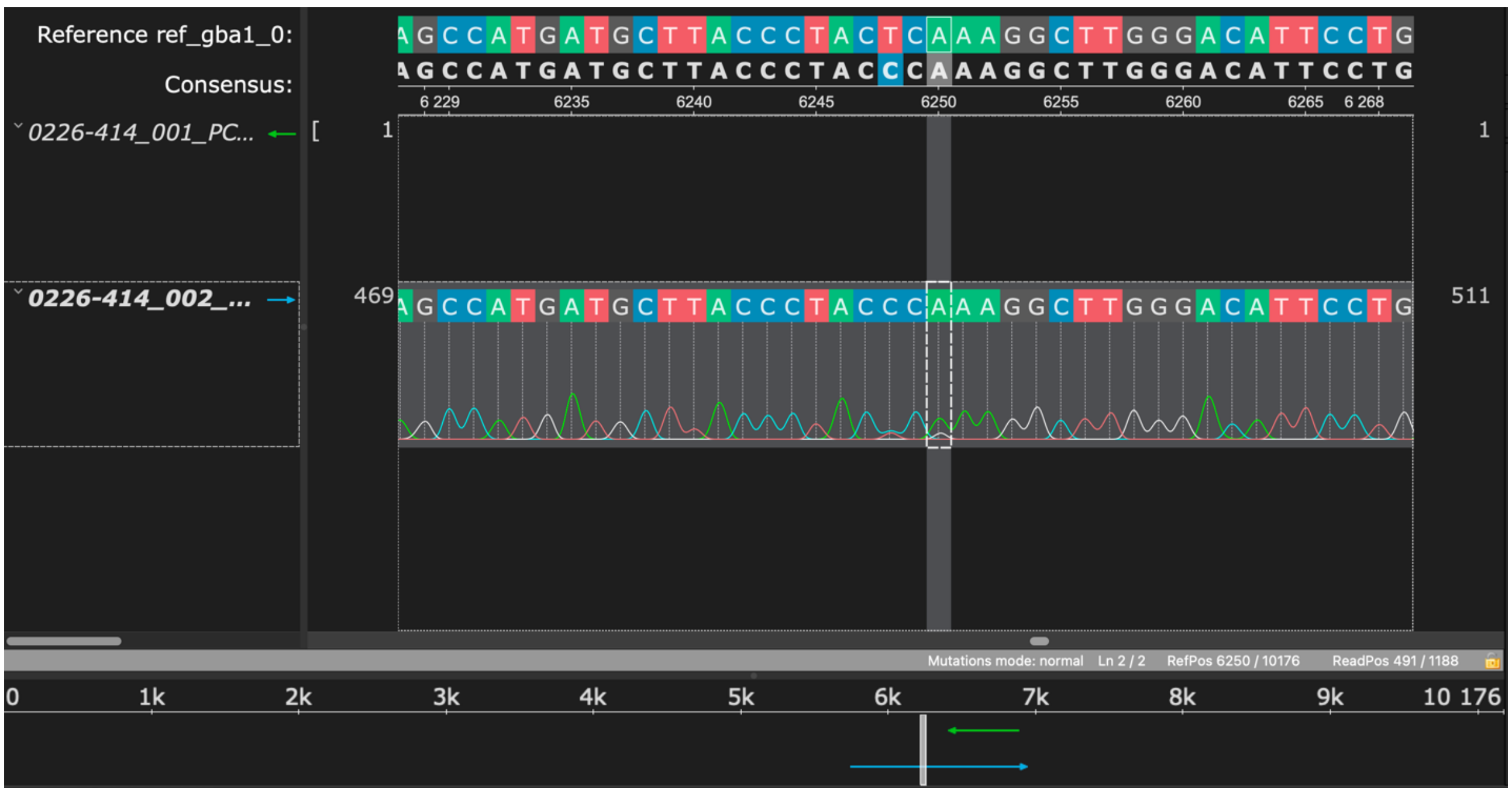

Figure S9. Sanger sequencing confirmation of the GBA1 variant in YLOPD20. Chromatogram viewed in IGV showing alignment of the Sanger sequencing read to the *GBA1* amplicon reference sequence (ref_gba1_0). The variant position (RefPos 6250, exon 2) is indicated by the vertical grey bar. Overlapping A and G signal peaks at this position confirm a heterozygous A>G substitution, concordant with the WGS genotype call.

IGV snapshots of haplotagged BAM files:

A)

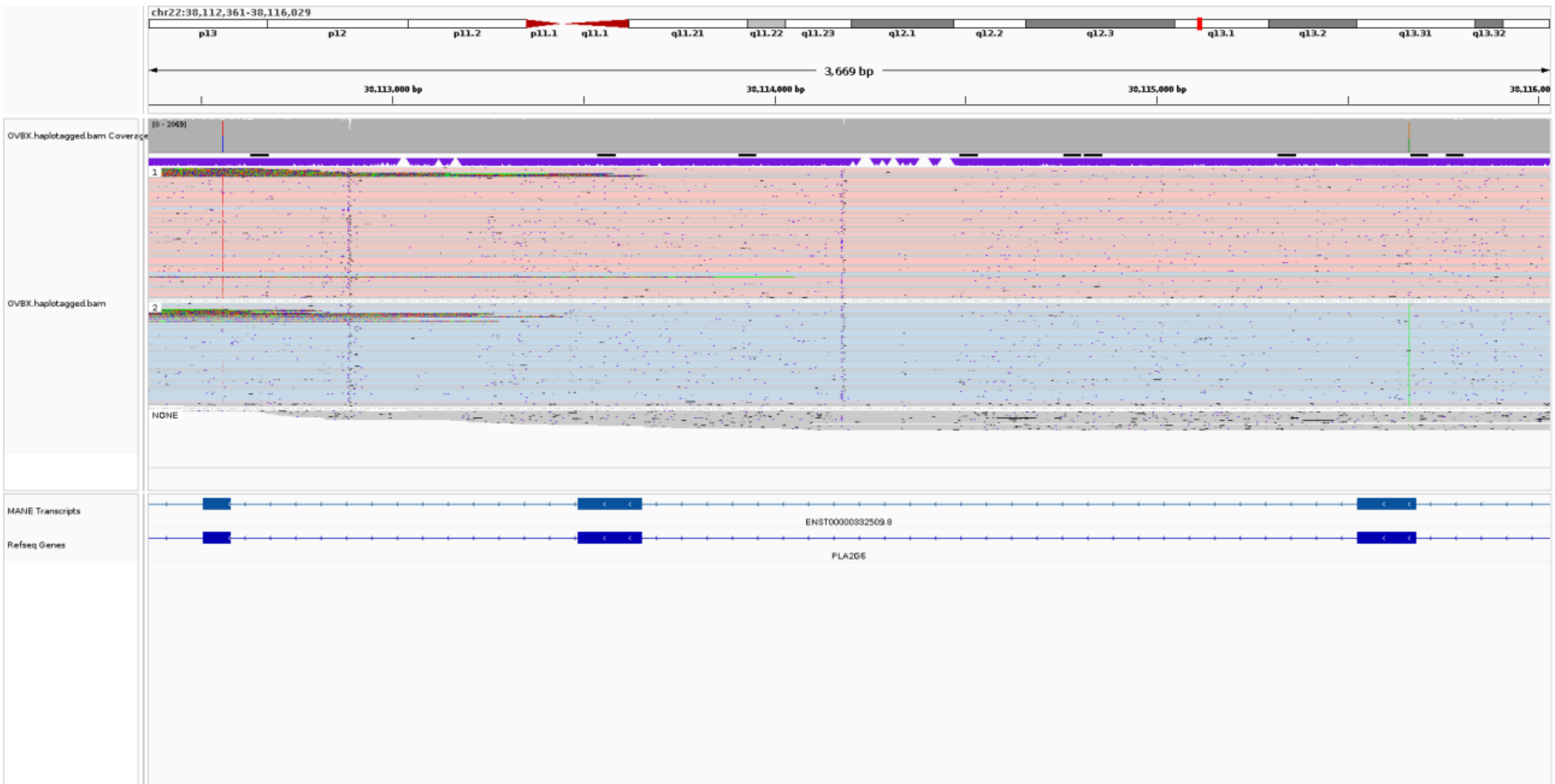

B)

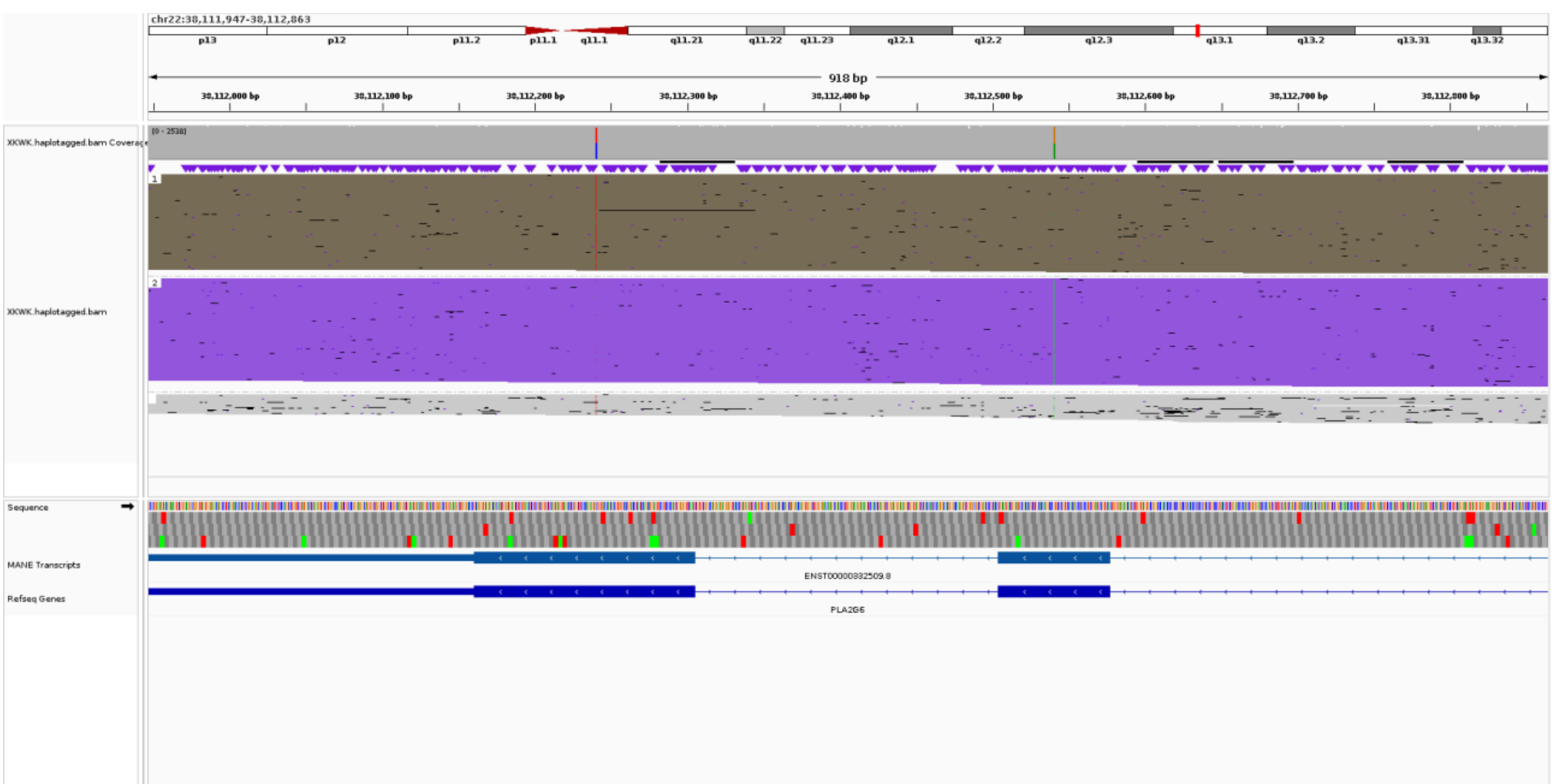

Figure S10. Haplotype-resolved IGV snapshots confirming compound heterozygosity in PLA2G6 gene. Haplotagged read alignments at the *PLA2G6* locus (chr22) for **(A)** YLOPD50

and **(B)** YLOPD238, phased using WhatsHap v2.3. Reads are grouped and coloured by haplotype tag (HP): YLOPD50 - HP1 (pink), HP2 (blue); YLOPD238 - HP1 (olive), HP2 (purple); unphased reads are shown in grey. Vertical coloured bars indicate the two variant positions in each sample. In both cases, each variant is carried exclusively on one haplotype, confirming that the two pathogenic variants are in trans (compound heterozygous configuration). YLOPD50: c.2222G>A (p.Arg741Gln) and c.1898C>T (p.Ala633Val). YLOPD238: c.2341G>A (p.Ala781Thr) and c.2239C>T (p.Arg747Trp). Mean sequencing depth was 264× (YLOPD50) and 213× (YLOPD238). Genomic coordinates are relative to GRCh38/hg38.

Burden testing, and PRS results:

Since YLOPD21, YLOPD21e, and YLOPD22 are siblings, we excluded the two affected siblings (YLOPD21 and YLOPD21e) and retained only YLOPD22. The rare variant burden test, performed using the CoCorV framework comparing cases against gnomAD SAS controls, yielded a genomic inflation factor of λemp = 1.02 (Figure S5). Similarly, PRS remained significantly elevated in cases relative to controls ($p = 4.92 \times 10^{-34}$) (FigureS6), consistent with results from the full cohort.

Supplementary Data Structure Description

Column name	Description
Variant_ID	**Unique identifier for each variant (formatted as chromosome:position:ref:alt).**
Sample_ID	**Internal ID of the sample carrying the variant.**
Gene	**Gene associated with the variant.**
Inheritance	**Mode of inheritance (e.g., AR, AD, X-linked).**
Type	**Variant type (e.g., missense, nonsense, splice-site, deletion).**
Genotype	**Zygosity of the variant in the sample (e.g., het/hom).**
Consensus_Genotype	**Consensus genotype derived from all five SV callers used for structural variant detection.**
Novel/Known	**Indicates whether the variant is novel or previously reported in databases such as ClinVar or gnomAD.**
ACMG	**Classification based on ACMG guidelines (P = Pathogenic, LP = Likely Pathogenic, VUS = Variant of Uncertain Significance, LB = Likely Benign, B = Benign).**
Evidences	**Supporting lines of evidence used for ACMG classification.**
FamilyHistory	**Presence or absence of family history of Parkinson's disease (PD).**
Freq	**Frequency within the study cohort.**
Control_count	**Number of occurrences in control samples.**
Phenotype	**Clinical category of the sample (e.g., YOPD, LOPD, HC).**
Canonical	**Indicates whether the variant affects the canonical transcript.**
Age	**Current age of the participant.**
AAO	**Age at onset of PD symptoms.**
Gender	**Participant sex (M or F).**
State	**State of origin of the participant.**
AAChange.ensGene	**Amino acid change annotation based on Ensembl transcript.**
AC	**Allele count observed in the dataset.**
AF	**Allele frequency observed in the dataset.**
AN	**Total number of alleles analyzed.**
CADD_phred	**CADD Phred-like score indicating predicted deleteriousness.**
CLNDISDB	**ClinVar disease database identifier(s).**
CLNDN	**ClinVar disease name(s).**
CLNREVSTAT	**ClinVar review status.**
CLNSIG	**ClinVar clinical significance.**
ClinPred_pred	**ClinPred prediction (D = Deleterious, T = Tolerated).**

Polyphen2_HVAR_pred	**PolyPhen-2 (HumVar) prediction (D = Probably damaging, P = Possibly damaging, B = Benign).**
Polyphen2_HVAR_rankscore	**PolyPhen-2 (HumVar) rank score.**
REVEL	**REVEL score for missense variant pathogenicity prediction.**
REVEL_rankscore	**Normalized rank score of REVEL.**
SIFT4G_converted_rankscore	**Normalized SIFT4G score.**
SIFT4G_pred	**SIFT4G prediction (D = Deleterious, T = Tolerated).**
AlphaMissense_pred	**AlphaMissense prediction (e.g., Pathogenic, Benign, Uncertain).**
AlphaMissense_pathogenicity_score	**Continuous pathogenicity score from AlphaMissense (0–1, higher = more pathogenic).**
gnomad40_genome_AF	**Allele frequency in gnomAD v4.0 (genome dataset).**
gnomad40_genome_AF_XX	**Allele frequency in female samples.**
gnomad40_genome_AF_XY	**Allele frequency in male samples.**
gnomad40_genome_AF_afr	**Allele frequency in African/African-American population.**
gnomad40_genome_AF_ami	**Allele frequency in Amish population.**
gnomad40_genome_AF_amr	**Allele frequency in Latino/Admixed American population.**
gnomad40_genome_AF_asj	**Allele frequency in Ashkenazi Jewish population.**
gnomad40_genome_AF_eas	**Allele frequency in East Asian population.**
gnomad40_genome_AF_fin	**Allele frequency in Finnish population.**
gnomad40_genome_AF_grpmax	**Highest observed allele frequency across all gnomAD populations.**
gnomad40_genome_AF_mid	**Allele frequency in Middle Eastern population.**
gnomad40_genome_AF_nfe	**Allele frequency in Non-Finnish European population.**
gnomad40_genome_AF_raw	**Raw allele frequency reported in gnomAD.**
gnomad40_genome_AF_remaining	**Allele frequency in unassigned or other populations.**
gnomad40_genome_AF_sas	**Allele frequency in South Asian population.**

Supplementary Data Legends

Data S1. Cohort Description

Data S2. List of 67 genes previously associated with PD, and 37 genes associated with dystonia and parkinsonism

Data S3. List of pathogenic variants in PD associated genes

Data S4. List of rare variants identified in 90 PD GWAS loci

Data S5. List of VUS in PD genes

Data S6. List of pathogenic variants in parkinsonism and dystonia genes

Data S7. List of all variants in parkinsonism and dystonia genes

Data S8. List of VUS in non-PD genes

Data S9. List of pathogenic variants in non-PD genes

Data S10. List of SVs in PD associated genes

Data S11. List of SVs in parkinsonism and dystonia genes

Data S12. List of SVs in non-PD genes

Data S13. PRS values

Data S14. Gene burden analysis results

Data S15. Pathway enrichment analysis results
